# Morphology of the papilla can predict procedural safety and efficacy of ERCP—a systematic review and meta-analysis

**DOI:** 10.1038/s41598-024-57758-9

**Published:** 2024-03-28

**Authors:** Edina Tari, Endre Botond Gagyi, Anett Rancz, Dániel Sándor Veres, Szilárd Váncsa, Péter Jenő Hegyi, Krisztina Hagymási, Péter Hegyi, Bálint Erőss

**Affiliations:** 1 Centre for Translational Medicine, Semmelweis University, Budapest, Hungary; 2 Institute of Pancreatic Diseases, Semmelweis University, Budapest, Hungary; 3 Selye János Doctoral College for Advanced Studies, Semmelweis University, Budapest, Hungary; 4 Department of Biophysics and Radiation Biology, Semmelweis University, Budapest, Hungary; 5 Institute for Translational Medicine, Medical School, University of Pécs, Pecs, Hungary; 6 Department of Surgery, Transplantation, and Gastroenterology, Semmelweis University, Budapest, Hungary

**Keywords:** Pancreatic disease, Therapeutic endoscopy, Gastroenterology

## Abstract

Endoscopic Retrograde Cholangiopancreatography (ERCP) is the primary therapeutic procedure for pancreaticobiliary disorders, and studies highlighted the impact of papilla anatomy on its efficacy and safety. Our objective was to quantify the influence of papilla morphology on ERCP outcomes. We systematically searched three medical databases in September 2022, focusing on studies detailing the cannulation process or the rate of adverse events in the context of papilla morphology. The Haraldsson classification served as the primary system for papilla morphology, and a pooled event rate with a 95% confidence interval was calculated as the effect size measure. Out of 17 eligible studies, 14 were included in the quantitative synthesis. In studies using the Haraldsson classification, the rate of difficult cannulation was the lowest in type I papilla (26%), while the highest one was observed in the case of type IV papilla (41%). For post-ERCP pancreatitis, the event rate was the highest in type II papilla (11%) and the lowest in type I and III papilla (6–6%). No significant difference was observed in the cannulation failure and post-ERCP bleeding event rates between the papilla types. In conclusion, certain papilla morphologies are associated with a higher rate of difficult cannulation and post-ERCP pancreatitis.

## Introduction

Endoscopic Retrograde Cholangiopancreatography (ERCP) is the most used therapeutic procedure for pancreaticobiliary disorders. However, how to best achieve safe and effective bile duct cannulation is still debated. Despite notable developments in the past decades, the failure rate is still 5–20% in experienced hands^1^. Moreover, the incidence of the procedure’s adverse events is high; post-ERCP pancreatitis (PEP) has an incidence rate of 9.7%, with a mortality rate of 0.7%^2^.

Endoscopists performing ERCP recognize the differences in the macroscopic appearance of the major papilla. This has led to a conception that certain appearances of the papilla are more challenging to cannulate and, therefore, more prone to adverse events. Despite the essential role of bile duct cannulation in procedural safety and success, research on this topic is still limited.

A Scandinavian research group published the first inter- and intraobserver-validated classification of the major papilla’s endoscopic appearance in 2017^3^. In the same year, they also published a multicentric prospective cohort study, indicating that the anatomy of the major papilla affects both the difficulty of the bile duct cannulation and the procedural adverse events^4^. Further, their results suggest that the morphology of the papilla should be considered in the training of fellow endoscopists^4^. Other identified studies support their results^5,6^.

Recently, several articles have been published assessing the influence of papilla morphology on ERCP outcomes, with contradicting results. Therefore, we aimed to systematically review and quantify the magnitude of its effect and investigate its importance and relevance in the endoscopic practice.

## Methods

A systematic review and meta-analysis were conducted following the Preferred Reporting Items for Systematic Review and Meta-Analysis (PRISMA) Statement (see Supplementary Table 12) and the recommendations of the Cochrane Handbook^7,8^. The review protocol was registered in advance on PROSPERO with the registration number CRD42022360894.

### Systematic search

Three databases: MEDLINE (via PubMed), Embase, and Cochrane Central Register of Controlled Trials (CENTRAL), were systematically searched from inception until the 29th of September 2022. We did not apply any filters or restrictions to our search. The main parts of the search query included terms in connection with ERCP and papilla morphology. For the detailed search strategy, see Table S1. Additionally, we systematically searched for relevant articles by reviewing the included articles’ bibliographic references and citation lists.

### Eligibility criteria

The condition-context-population (CoCoPop) framework was used to identify eligible studies^9^. The conditions were (Co): difficult cannulation, cannulation attempts, cannulation time, cannulation failure, post-ERCP pancreatitis, and other post-ERCP adverse events (bleeding, perforation, infection) in the context of the different papilla morphologies (Co). Studies with adult patients (> 18) undergoing ERCP with a native papilla (Pop) were selected.

Randomized controlled trials, case–control, cross-sectional, and cohort studies were eligible for inclusion. Both full-text articles and conference abstracts with sufficient data were considered eligible. Regarding the definition of difficult cannulation, cannulation failure, and post-ERCP adverse events, the definitions provided in the included studies were used.

### Morphology of the papilla

Primarily, for the classification of the morphology of the papilla, as the first validated intra- and interobserver classification, the Haraldsson system was used^4^. They classified the papilla into four types: regular (type 1), small (type 2), protruding or pendulous (type 3), and creased or ridged (type 4)^3^.

Secondarily, a comparison between the Haraldsson and the other identified classification systems was attempted with the following method: two endoscopists (PJH, EB) assessed the description of the morphology and the imagery of the studies. They chose the identical papilla types to Haraldsson’s. In case of any disagreement, a third reviewer was included in the decision process (ET). After the comparison, additional analyses were conducted.

### Study selection and data extraction

After the systematic search, the yielded articles were imported into a reference management program (EndNote X7.4, Clarivate Analytics, Philadelphia, PA, USA) to remove the duplicates automatically and manually. After removing duplicates, two independent authors (ET, EBG) screened the remaining publications first by title and abstract and then by full text. We used Rayyan for the selection process^10^. Cohen’s kappa coefficient (κ) was calculated on both levels of selection to measure inter-reviewer reliability^11^.

Two investigators extracted data independently (ET, EBG) and manually populated it into a purpose-designed Excel 2016 sheet (Office 365, Microsoft, Redmond, WA, USA). Data were collected on the first author, year of publication, digital object identifier, period of data collection, study location, number of centers, study design, the mean or median age of the patients (with standard deviation or interquartile range), the total number of patients, the number of women, the number of patients with each papilla morphology, and data regarding the primary and secondary outcomes in the context of the different papilla types. For statistical analysis, raw data were extracted into two-by-four tables (condition yes/no; papilla morphologies).

### Statistical analysis

The statistical analysis was performed by a biostatistician (DSV) with *R* (R Core Team 2022, v4.2.2)^12^. Forest plots were used to display the results of the meta-analytical calculations. The minimum study number to perform the meta-analytical calculation were three. Event rates with a 95% confidence interval (CI) were used for the effect size measure. As we anticipated considerable between-study heterogeneity, a random-effects model was used to pool effect sizes. For assessing the small study publication bias, funnel plots were used with a visual inspection. Additional sensitivity analyses were conducted using the leave-one-out method, with a minimum study number of four (see additional details in the supplementary material).

See supplementary material for additional details on the statistical analyses.

### Risk of bias assessment

Two investigators (ET, EBG) independently assessed the risk of bias for each outcome using the Joanna Briggs Institute Critical Appraisal tool for studies reporting prevalence^13^.

### Quality of evidence

Certainty of evidence was assessed following the Grading of Recommendations Assessment, Development, and Evaluation (GRADE) recommendation^14^. Two independent investigators (ET, EBG) evaluated all criteria for all outcomes. Disagreements were resolved by the senior review author (BE).

## Results

### Search and selection

**Figure 1:**
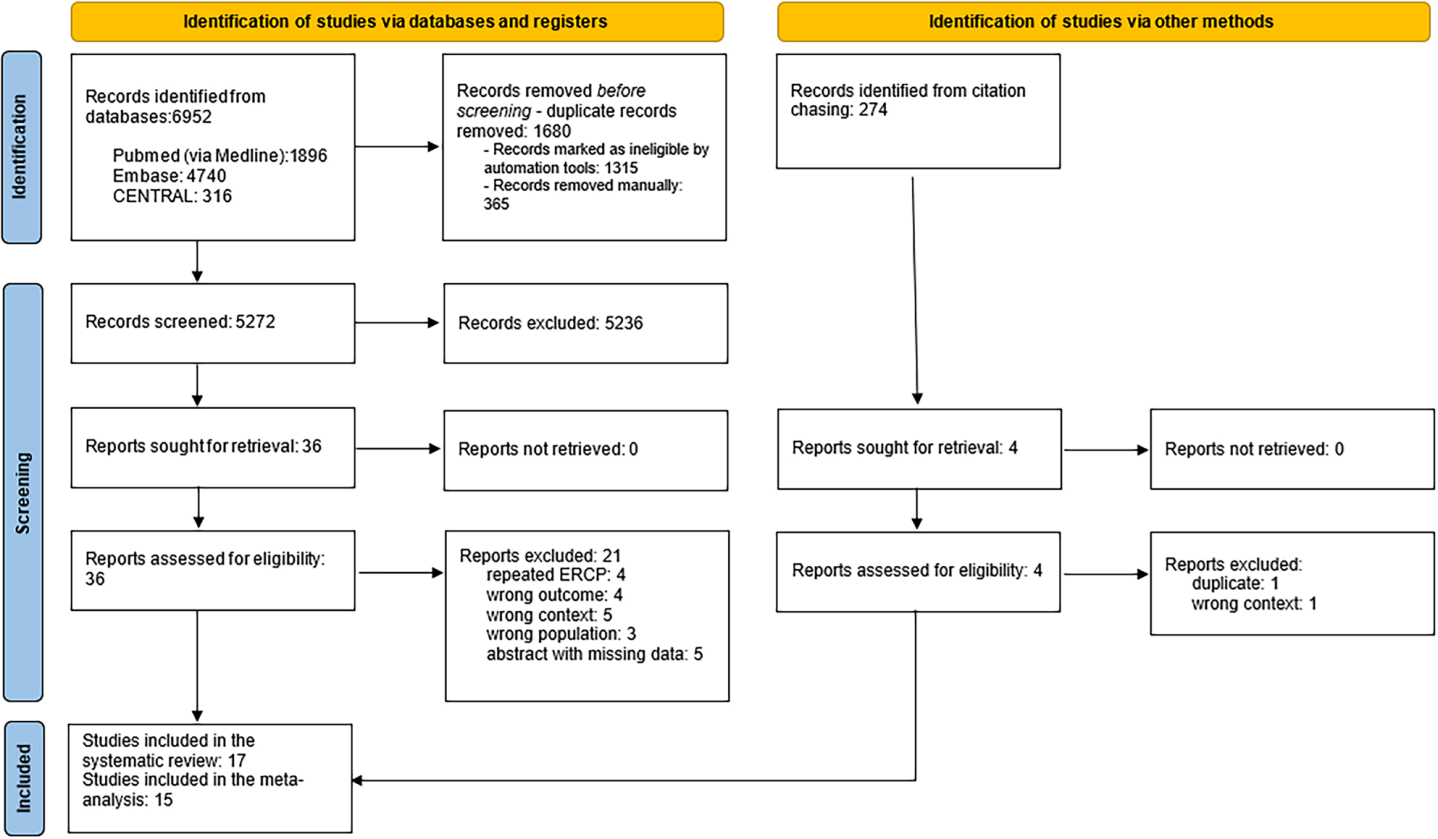
PRISMA 2020 flowchart representing the study selection process.

The details of the study selection process are summarised in the PRISMA flow chart shown in Fig. 1.

A total of 6,952 studies were identified through database searching. Finally, our narrative synthesis comprised 17 studies^4–6,15–28^. Of those, 14 could be included in the quantitative synthesis^4–6,15–17,19,21–27^.

### Basic characteristics of included studies

The main characteristics of the included studies are summarised in Table 1. Eligible studies were reported between 2016 and 2022. Of the 17 studies, 15 were cohort studies, eight had prospective (5, 6, 19–22, 26, 27), and seven had retrospective designs. There was also one case–control^24^ and one cross-sectional study^19^. 13 of the studies were full-text articles^4–6,15–17,19,21,22,24,26–28^, and four of them were conference abstracts^18,21,23,25^. Seven studies used the Haraldsson classification^4–6,19,22,24,25^, with seven additional ones using comparable classifications^15–17,21,23,26,27^. Three studies used classification systems that were not comparable to the Haralddson classification. The number of study participants ranged from 72 to 11,090.

**Table 1:** Basic characteristics of included studies.

Author	Year	Country	Centers	Study type	Study period	Age (*:mean; #:median)	Sex (female %)	Number of patients	Classification	Outcomes
Balan et al.^15^	2020	Romania	1	Prospective cohort	January 2018 to August 2018	NA	NA	322	Regular: 52% Canard type I 11%: Canard type II: 19% Canard type III: 10% Canard type IV: 8%	Difficult cannulation Cannulation time Cannulation attempts Post-ERCP pancreatitis Post-ERCP bleeding Post-ERCP infection
Canena et al.^16^	2021	Portugal	3	Prospective cohort	May 2018 to October 2020	*69.6	56.8%	361	Viana type I: 13% Viana type IIa: 35% Viana type IIb: 30% Viana type IIc:10% Viana type IIIa: 4% Viana type IIIb: 4% Viana type IV: 4%	Cannulation failure Cannulation time Post-ERCP pancreatitis Post-ERCP bleeding Post-ERCP perforation
Chen et al.^5^	2020	Taiwan	1	Prospective cohort	October 2017 to October 2018	*64 (SD: 16.5)	47.5%	286	Haraldsson type I: 41% Haraldsson type II: 9% Haraldsson type III: 22% Haraldsson type IV: 28%	Cannulation failure Cannulation time Post-ERCP pancreatitis Post-ERCP bleeding Post-ERCP perforation Post-ERCP cholangitis
Fernandes et al.^18^	2018	Portugal	3	Prospective cohort	August 2017 to January 2018	#79	59.4%	106	Leés type I: 50% Leés type II: 32% Leés type III: 12% Leés type IV: 6%	Cannulation time
Gutierrez- De Aranguren et al.^19^	2021	Peru	1	Retrospective cross-sectional	July 2019 to April 2021	*55 (SD:2 0)	66.5%	188	Haraldsson type I: 32% Haraldsson type II: 25% Haraldsson type III: 27% Haraldsson type IV: 16%	Difficult cannulation
Haraldsson et al.^4^	2019	Nordic countries	9	Prospective cohort	NA	66 (SD: 16)	52%	1377	Haraldsson type I: 56% Haraldsson type II: 13% Haraldsson type III: 23% Haraldsson type IV: 8%	Difficult cannulation Cannulation time Post-ERCP pancreatitis
Liu et al.^20^	2021	China	1	Retrospective cohort	January 2008 to December 2017	NA	NA	11 090	Normal: 44% Thick and long: 11%: Peridiverticular: 27% Intradiverticular: 5% Ectopic: 1% Edematous 10%: Ulcerative: 2%	Difficult cannulation
Mohamed et al.^6^	2021	Canada	1	Retrospective cohort	September 2018 to January 2020	NA	51.8%	637	Haraldsson type I: 62% Haraldsson type II: 5% Haraldsson type IIIa: 9% Haraldsson type IIIb: 9% Haraldsson type IV: 3% Type D: 12%	Cannulation failure Cannulation time Cannulation attempts Post-ERCP pancreatitis Post-ERCP bleeding Post-ERCP infection Post-ERCP cholangitis or sepsis
Nakeeb et al.^17^	2016	Egypt	1	Prospective cohort	August 2012 to September 2014	*58.4 (SD: 14.7)	44.4%	996	Normal: 60% Atrophic: 3% Pregnant: 7% Tumor: 7% Redundant: 8% Juxtadivertcular: 8% Small: 6% Long: 1%	Post-ERCP pancreattis
Onilla et al.^21^	2021	Philippines	1	Retrospective cohort	January 2017 to December 2019	NA	NA	347	Regular protrusion: 57% Small protrusion: 31% Large protrusion: 12% Annular pattern: 72% Unstructured pattern: 11% Longitudinal pattern 11%: Isolated pattern: 1% Gyrus pattern: 5%	Difficult cannulation Cannulation failure
Quiroga-Purizaca et al.^22^	2022	Peru	1	Propective cohort	NA	*51.5 ( CI 48.8–54.1)	68.4%	138	Haraldsson type I: 59% Haraldsson type II: 8% Haraldsson type III: 29% Haraldsson type IV: 4%	Difficult cannulation Cannulation time Cannulation attempts Post-ERCP pancreatitis Post-ERCP bleeding Post-ERCP perforation
Sadeghi et al.^23^	2019	Iran	1	Prospective cohort	September 2017 to March 2018	*62.3 (SD: 15.5)	51.4%	72	Small: 33%: Bulging: 28% Long: 39%	Cannulation success
Saito et al.^24^	2022	Japan	3	Retrospective case–control	April 2012 to February 2020	*74.9	47.5%	1406	Haraldsson type I: 45% Haraldsson type II: 44% Haraldsson type III: 7% Haraldsson type IV: 4%	Difficult cannulation
Thongsuwan et al.^25^	2021	Thailand	1	Retrospective cohort	January 2013 to May 2017	NA	50.4%	558	Haraldsson type I: 66% Haraldsson type II: 16% Haraldsson type III: 12% Haraldsson type IV: 6%	Difficult cannulation Cannulation failure Post-ERCP pancreatitis, Post-ERCP bleeding Post-ERCP infection
Watanabe et al.^26^	2019	Japan	1	Retrospective cohort	September 2013 to June 2017	#70	36%	589	Regular protrusion: 12% Small protrusion: 78% Large protrusion: 10% Annular pattern: 67% Unstructured pattern: 7% Longitudinal pattern: 7% Isolated pattern: 1% Gyrus pattern:16% Unclassified pattern: 2%	Difficult cannulation Cannulation failure Cannulation attempts
Zhang et al.^27^	2016	China	1	Retrospective cohort	February 2012 to March 2015	*75 (SD: 2.2)	42.7%	82	bulging: 44% normal: 22% small: 16% unusual location: 18%	Cannulation failure Cannulation time
Zheng et al.^28^	2020	China	1	Retrospective cohort	January 2016 to December 2019	NA	46.1%	2385	others:18% villous: 74% granular: 8%	Post-ERCP pancreatitis

### Quantitative synthesis

#### Difficult cannulation

**Figure 2:**
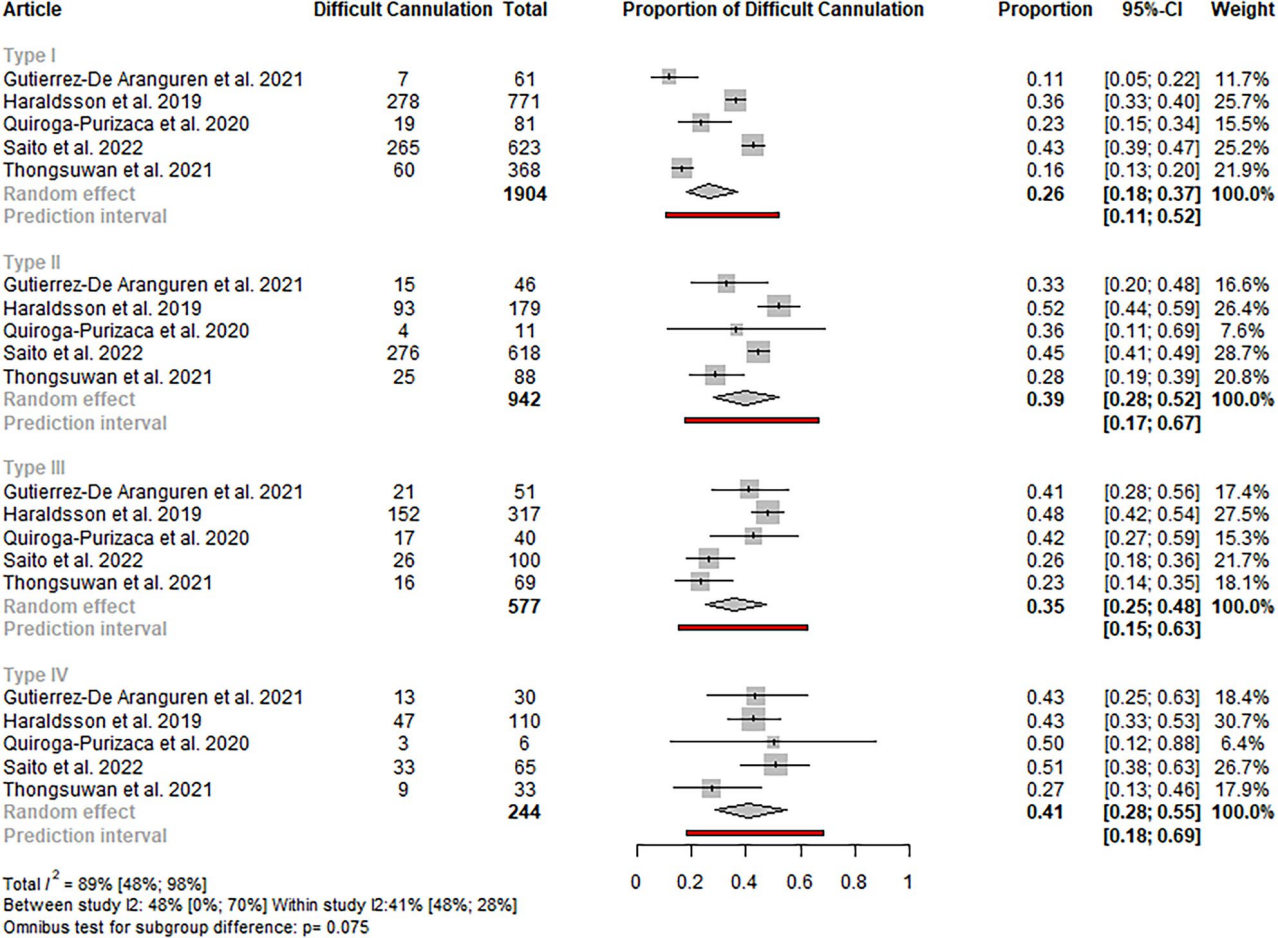
Forest plot representing the pooled event rate of difficult cannulation in the different papilla types in studies using the Haraldsson classification, showing a lower tendency for difficult cannulation in type I papilla compared to the other papilla types.

Nine studies were identified regarding the event rate of difficult cannulation^4,15,19–22,24–26^, of which eight were included in the quantitative synthesis^4,15,19,21,22,24–26^. In the case of studies using the classification proposed by Haraldsson, in type I papilla, the rate of difficult cannulation was lower (26%; CI 18–37) compared to the other papilla types (type III: 35%; CI 25–48; type II: 39%; CI 28–52; type IV: 41%; CI 28–55). The difference was statistically no significant; however, the p-value referred for a higher tendency for difficult cannulation in certain papilla types (p: 0.075). The heterogeneity was high (total I^2^: 89%; CI 48–98). Sensitivity analyses did not reveal outlier studies or relevant changes in the estimate (see Figs. 2 and S1).

A similar but statistically significant result with no outlier study was observed, including all the studies with different classifications (p: 0.019; total I^2^: 87%; CI 55**–**96) (see Figures S2-3).

#### Cannulation failure

**Figure 3:**
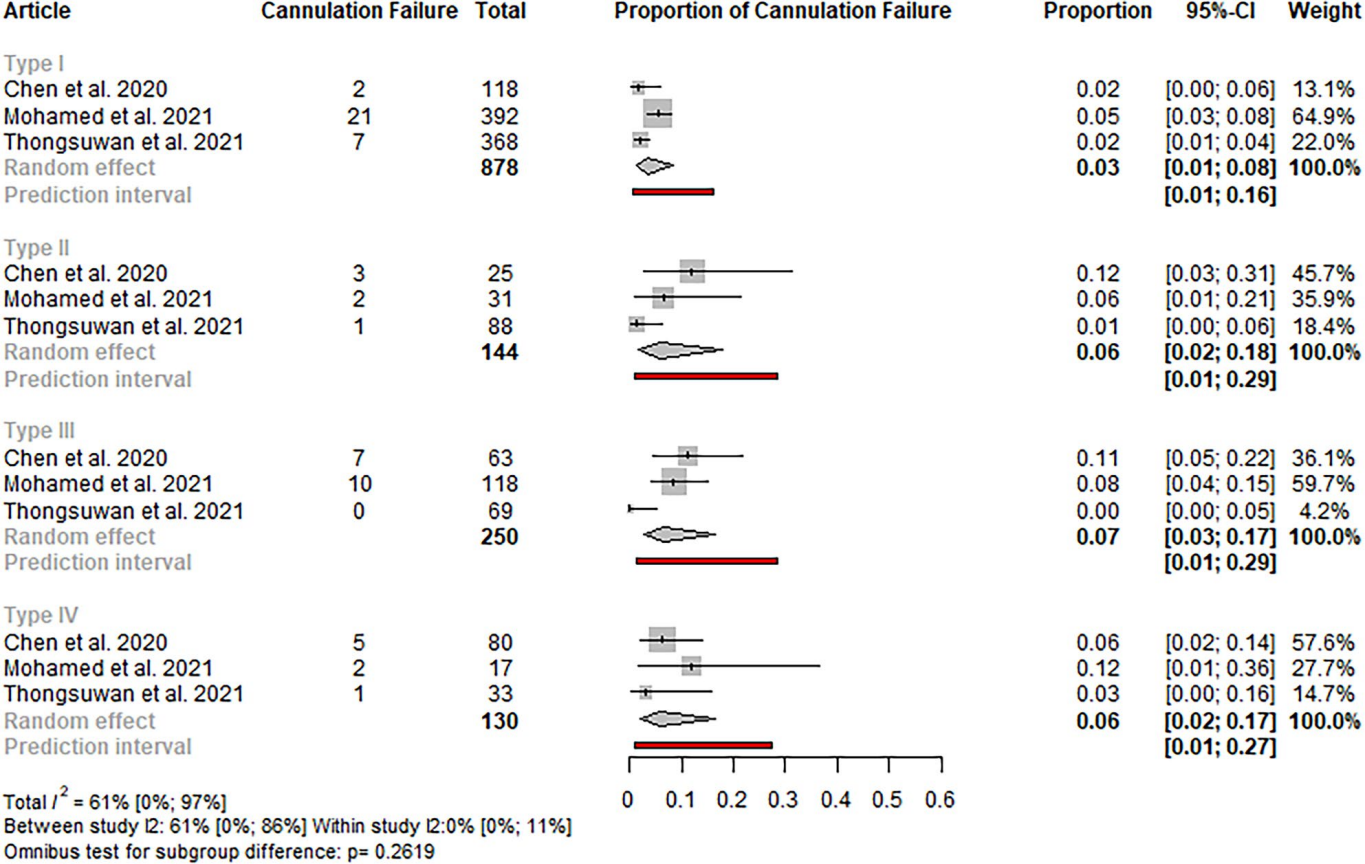
Forest plot representing the pooled event rate of cannulation failure in the different papilla types in studies using the Haraldsson classification, showing no statistically significant difference in the event rates between the papilla types.

Eight studies detailed the event rate of cannulation failure, all using Haraldsson’s or classifications comparable to it^5,6,16,21,23,25,26,28^. In the analysis, including studies only using the Haraldsson classification, no statistically significant difference was observed in the rate of failed cannulation between the different papilla types (p: 0.262, total I^2^: 61%; CI 0**–**97) (see Fig. 3).

In the case of including all eight studies, the difference was statistically significant (p: 0.047, I^2^: 64%; CI 0**–**91). The rate of cannulation failure was the highest in the case of type II papilla (8%, CI 4–14) and the lowest in type I (3%; CI 2–6) (see Figure S4). Sensitivity analyses did not reveal outlier studies or relevant changes in the estimate (see Figure S5).

#### Post-ERCP pancreatitis

**Figure 4:**
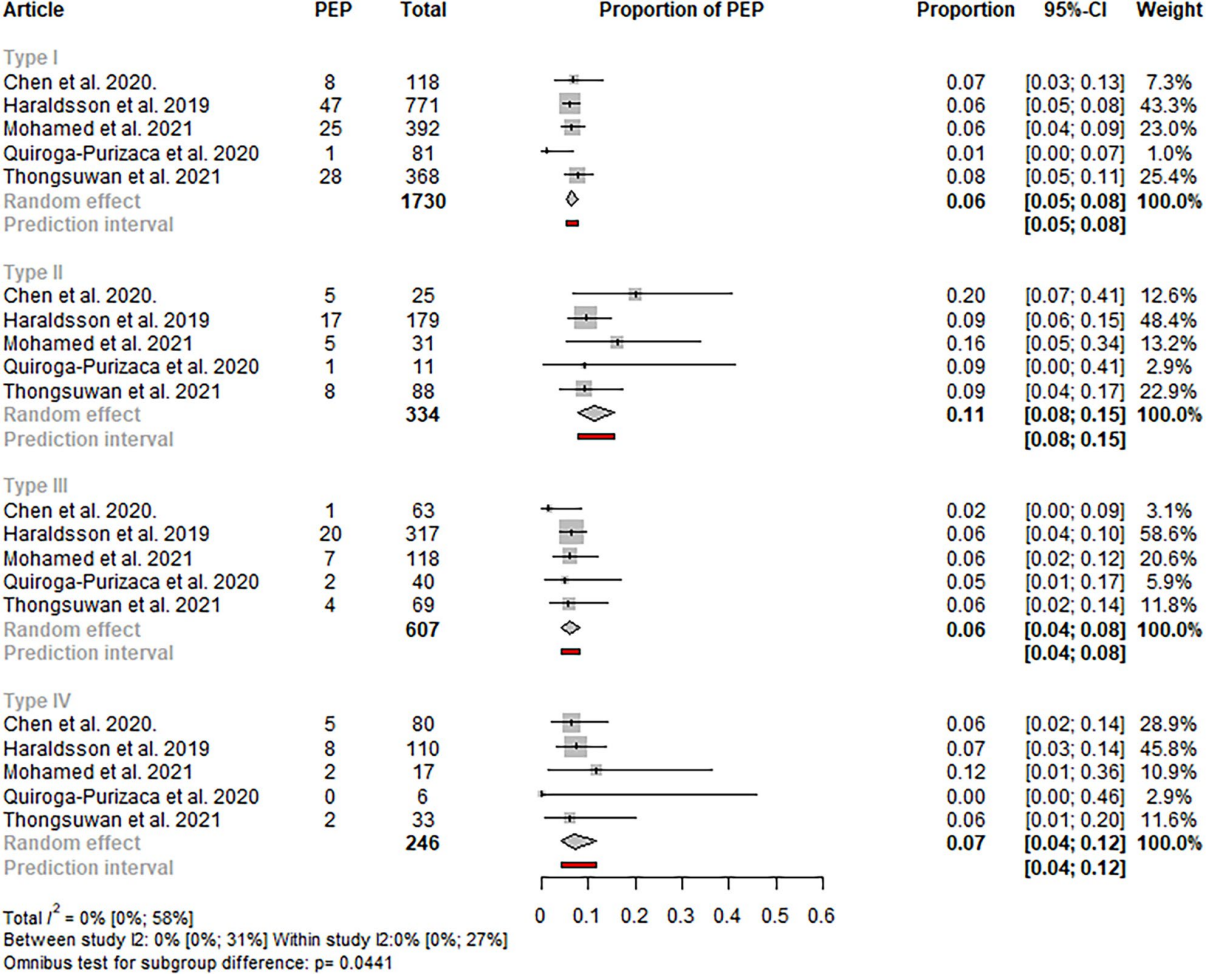
Forest plot representing the pooled event rate of post-ERCP pancreatitis in the different papilla types in studies using the Haraldsson classification, showing a statistically significantly higher rate of post-ERCP pancreatitis in type II papilla, compared to the other papilla types.

Nine of the identified studies reported the event rate of PEP in the different papilla types^4–6,15–17,22,25,28^, of which eight articles were included in the quantitative synthesis^4–6,15–17,22,25^. In the case of studies using the Haraldsson classification, in type II papilla, the rate of post-ERCP pancreatitis was higher (11%; CI 8**–**15) compared to the other papilla types (type IV: 7%; CI 4**–**12; type I: 6%; CI 5**–**8; type III: 6%; CI 4**–**8). The result was statistically significant (p: 0.0441). Total homogeneity was observed (total I^2^: 0.044) (see Fig. 4).

A similar tendency was observed in the case of including all eight studies; however, the difference between the papilla types was not statistically significant (p: 0.103) (see Figure S6). Sensitivity analyses did not reveal outlier studies or relevant changes in the estimate (see Figures S7-8).

#### Post-ERCP bleeding

**Figure 5:**
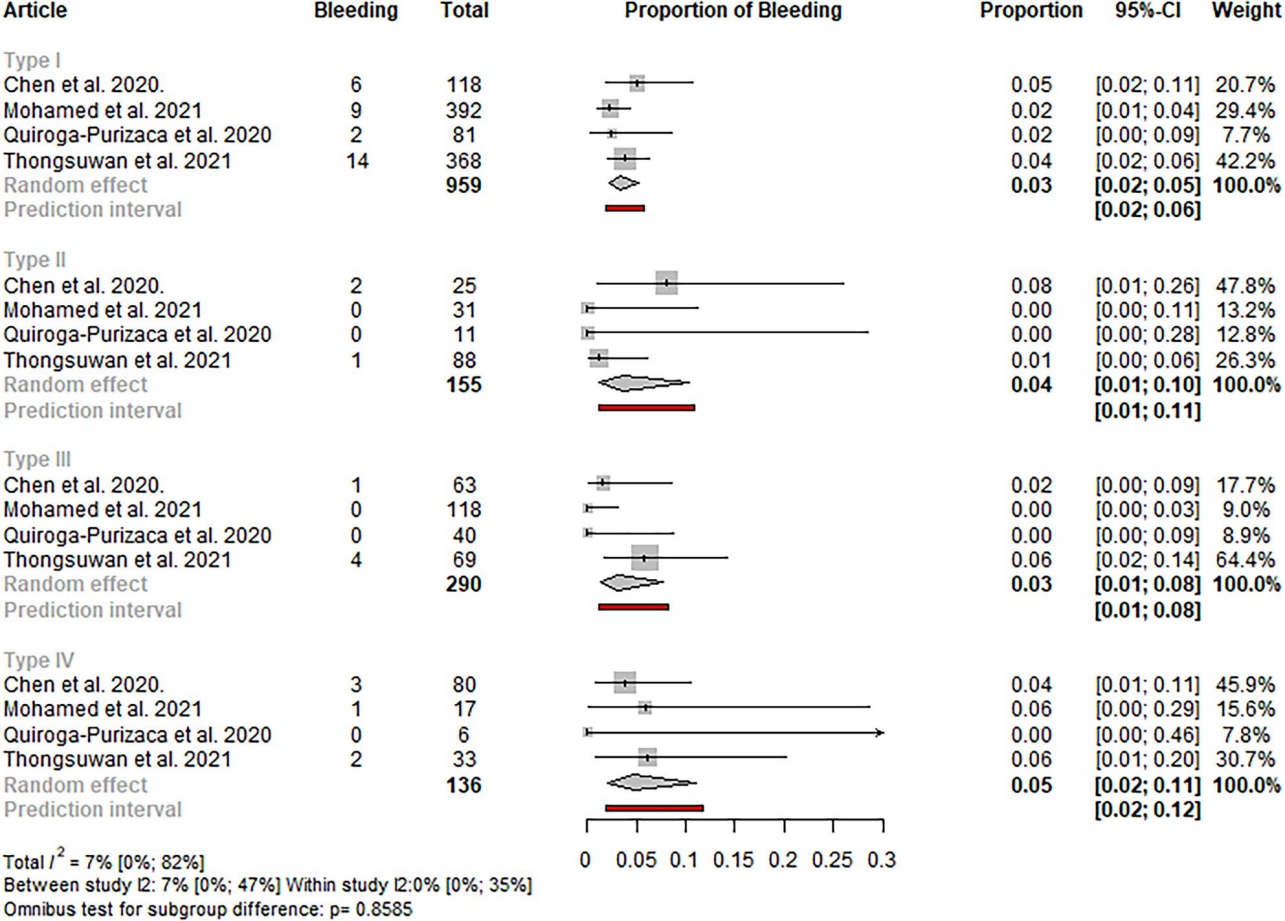
Forest plot representing the pooled event rate of post-ERCP bleeding in the different papilla types in studies using the Haraldsson classification, showing no statistically significant difference in the event rates between the papilla types.

Six eligible studies reported information about a bleeding episode after an ERCP procedure, all using the Haraldsson classification or classifications comparable to it^5,6,15,16,22,25^. In the analyses with only studies using the Haraldsson classification and with all classification systems, no statistically significant difference was observed in the event rate of the post-ERCP bleeding between the papilla types (p: 0.8585 and p: 0.8078, respectively) (see Figs. 5 and S9). Sensitivity analyses did not reveal outlier studies or relevant changes in the estimate (see Figures S10-11).

### Qualitative synthesis

#### Cannulation time

Eight studies investigated cannulation time in the context of papilla morphology^4–6,15,16,18,22,27^, and four used the Haraldsson classification^4–6,22^. The time for cannulation was the lowest in type I papilla, without exception. Two-two studies reported the highest cannulation time in type II^4,5^ and type IV papilla^6,22^.

#### Cannulation attempts

Four studies investigated the number of cannulation attempts in the context of papilla morphology^6,15,22,26^, from which two used the Haraldsson classification^6,22^. In both cases, the cannulation attempts were the highest in type IV and the lowest in type I and III papillae.

#### Post-ERCP perforation

Three studies investigated the perforation rate after an ERCP procedure, all using the Haraldsson classification^5,16,22^. The meta-analytical calculation was impossible due to the number of zero events.

#### Post-ERCP infection

Four studies reported the proportion of patients with an infection after ERCP^5,6,15,25^; of those, three studies used the Haraldsson classification^5,6,25^. Chen et al. reported the highest event rate of cholangitis in type I (2.5%) and no event in type II and III papillae^5^. Mohammed et al. found the highest event rate of cholangitis and/or sepsis in type II (3.2%) and no event in type III and IV papillae, meanwhile in the study by Thongsuwan et al., the event rate of infection was the highest in type III (10.5%) and the lowest in type I papilla (6%)^6,25^.

### Risk of bias and publication bias assessment

Most of the included studies carried a low risk of bias. Among the eight studies detailing difficult cannulation, two (25%) had high, and six (75%) had low risk of bias. The results of the risk of bias assessments are shown in Figures S12-19. Publication bias could not be observed in the conducted analyses. The results of the assessments are shown in Figures S20-27.

### Quality of evidence

Since we included only cohort studies, the certainty of evidence ranged between very low and low for each outcome. Detailed results of the GRADE assessment can be found in Tables S4-11.

## Discussion

Our systematic review and meta-analysis assessed the impact of papilla morphology on ERCP and its outcomes. We found that in studies using the Haraldsson classification, compared to the other papilla types, the event rate of difficult cannulation was lower in type I papilla. Type II papilla was associated with a twofold increase in the event rate of PEP compared to the other papilla types. There was no difference in the cannulation failure and post-ERCP bleeding event rates between the different papilla types.

Since its introduction, there have been debates regarding ERCP’s safety and success rate. Several factors seem to influence cannulation difficulties, such as age and age-related factors, including duodenal distortion; procedure-related aspects, such as duodenal positioning or certain etiologies, for example, malignant biliary obstruction. The morphology of the papilla is also assumed to be related to multiple perspectives of the procedure^29^.

First, papilla morphology should be considered in the training of fellow endoscopists. In the studies selected for inclusion, there are contradicting data regarding how the endoscopist’s expertise influences cannulation difficulty. Mohamed et al. found no relationship between the rate of difficult cannulation and the endoscopist’s expertise (7). In contrast, in the study by Haraldsson et al., the rate of difficult cannulation was the highest in type II papilla, where the number of trainees starting the cannulation process was the highest (5). Other studies also suggest that the operator’s experience may decrease the rate of difficult cannulation and cannulation failure (34, 35). Further data in the literature suggest that the rate of PEP and other adverse events also decreases with the endoscopist’s experience (36).

Secondly, papilla morphology also influences the rate of PEP, the procedure’s most common adverse event^2^. We found the highest rate of PEP in type II papilla, which is consistent with the result of the individual studies. However, the definite explanation for this pattern is still uncertain. According to Chen et al. hypothesis, it could be due to the fact that endoscopic papilla balloon dilatation (EPBD) was used more often in this papilla type in their cohort^5^. The same trend could be observed in the study by Mohamed et al.^6^. Further data in the literature suggest that EPBD with small-caliber balloons (diameter: 8–10 mm) increases the rate of PEP^30^.

Lastly, all the included studies observed differences in rescue techniques’ use in different papilla morphologies. It could be one of the explanations for the non-significant difference in cannulation failure between the different papilla types. We hypothesize that the morphology of the papilla should be considered when choosing a rescue cannulation technique since it decreases the difference in the tendency for cannulation failure or difficult cannulation between the papilla types. Studies suggest that a pre-cut sphincterotomy or needle-knife fistulotomy (NKF) may be used in normal papillae. Trans-pancreatic sphincterotomy could be the recommended rescue technique in small papillae. In protruding/pendulous or creased/ridged papillae, also NKF could be the preferred method^31,32^.

Several classification systems were identified; the Haraldsson was the most widely used and well-recognized one. Despite being the first validated classification system developed by expert endoscopists and, therefore, the basis of our analysis, it has one major limitation: it ignores the presence of a periampullary diverticulum. A modified version of the classification was proposed by Mohamed et al. in 2021, introducing an additional papilla type (type D) for papillae involved with a periampullary diverticulum^6^. In addition, a meta-analysis by Mui et al. found that the presence of PAD may increase the risk of cannulation failure and may also be associated with a higher risk for post-ERCP adverse events^33^. These results suggest that this modified version of the classification should be used.

### Strengths

Despite the topic’s importance, to our knowledge, this is the first meta-analysis focusing on papilla morphology and its relation to the most relevant endpoints of the ERCP cannulation process and the rate of adverse events. A rigorous methodology was applied, with a comprehensive search key. No publication bias or outlier study was detected in any conducted analyses, and most studies carried a low risk of bias. Moreover, the number of included patients was above 20,000.

### Limitations

Regardless of all the strengths, this study also had some limitations: (1) In certain analyses, considerable statistical heterogeneity was observed. Its explanation could be the clinical heterogeneity across studies, such as the difference in the applied definitions in connection with the endoscopic procedure. Most studies used the definition of the European Society of Gastrointestinal Endoscopy for difficult cannulation; however, Thongsuwan et al. used its simplified version. (2) Some of the included cohort studies were retrospective analyses. (3) The certainty of the evidence was low or very low. (4) Abstracts were also eligible for inclusion; however, all were high-quality, containing all the necessary data.

### Implication for practice

Based on our results, during training of fellow endoscopists**,** papilla morphology should be determined, and trainees should start their learning with type I (“regular”) papillae. Using a unified classification system for papilla morphology is recommended to promote transparency in clinical practice.

### Implication for research

Large sample cohorts are needed to validate the Mohammed version of the classification and assess the presence of a periampullary diverticulum. Besides the event rate, future research should also focus on the severity of PEP in the different papilla types. Furthermore, developing a recommendation system for advanced cannulation techniques in the context of papilla morphologies should be considered.

## Conclusion

In conclusion, other types are associated with a higher rate of difficult cannulation compared to the regular papilla type. The small papilla is associated with a higher rate of post-ERCP pancreatitis.

### Supplementary Information

Supplementary Information 1.

## Data Availability

All data is provided within the manuscript or supplementary information files.
