# Supplementary material for: Morphology of the papilla can predict procedural safety and efficacy of ERCP—a systematic review and meta-analysis
Source: Sci Rep. 2024 Mar 28;14:7341. doi: 10.1038/s41598-024-57758-9 (PMC10973369; doi:10.1038/s41598-024-57758-9)
Supplement: Supplementary file 1 — Supplementary Information 1. [file 41598_2024_57758_MOESM1_ESM.docx]

**Title**

Morphology of the papilla can predict procedural safety and efficacy of ERCP – a systematic review and meta-analysis

**Authors**

Edina Tari^1,2^, Endre Botond Gagyi^1,3^, Anett Rancz^1^, Dániel Sándor Veres^1,4^, Szilárd Váncsa^1,2,5^, Péter Jenő Hegyi^1,2,5^, Krisztina Hagymási^1,6^, Péter Hegyi^1,2,5^, Bálint Erőss^1,2,5*^

**Affiliations:**

1. Centre for Translational Medicine, Semmelweis University, Budapest, Hungary
2. Institute of Pancreatic Diseases, Semmelweis University, Budapest, Hungary
3. Selye János Doctoral College for Advanced Studies, Semmelweis University, Budapest
4. Department of Biophysics and Radiation Biology, Semmelweis University, Budapest, Hungary
5. Institute for Translational Medicine, Medical School, University of Pécs, Pécs, Hungary
6. Department of Surgery, Transplantation, and Gastroenterology, Semmelweis University, Budapest, Hungary

**Contact information:**

Edina Tari: [edina.tari@gmail.com](mailto:edina.tari@gmail.com)

Endre Botond Gagyi: [endre.gg@gmail.com](mailto:endre.gg@gmail.com)

Anett Rancz: [ranczanett@gmail.com](mailto:ranczanett@gmail.com)

Dániel Sándor Veres: [daniel.s.veres@gmail.com](mailto:daniel.s.veres@gmail.com)

Szilárd Váncsa: [vancsa.szilard@gmail.com](mailto:vancsa.szilard@gmail.com)

Péter Jenő Hegyi: [drdunajkastreda@gmail.com](mailto:drdunajkastreda@gmail.com)

Krisztina Hagymási: [hagymasi.krisztina@med.semmelweis-univ.hu](mailto:hagymasi.krisztina@med.semmelweis-univ.hu)

Péter Hegyi: [hegyi2009@gmail.com](mailto:hegyi2009@gmail.com)

Bálint Erőss: [dr.eross.balint@gmail.com](mailto:dr.eross.balint@gmail.com)

**FIGURE LEGENDS**

**Supplementary Figure 1.** Forest plot representing the influential analysis with the leave-one-out method in the outcome of difficult cannulation in studies using the Haraldsson classification

**Supplementary Figure 2.** Forest plot representing the pooled event rate of difficult cannulation in the different papilla types in studies using different classification systems, showing statistically significantly lower rate in type I papilla, compared to the other papilla types

**Supplementary Figure 3.** Forest plot representing the influential analysis with the leave-one-out method in the outcome of difficult cannulation in studies using different classification systems

**Supplementary Figure 4.** Forest plot representing the pooled event rate of cannulation failure in the different papilla types in studies using different classification systems, showing statistically significant difference in the event rates between the papilla types

**Supplementary Figure 5.** Forest plot representing the influential analysis with the leave-one-out method in the outcome cannulation failure in studies using different classification systems

**Supplementary Figure 6.** Forest plot representing the pooled event rate of post-ERCP pancreatitis in the different papilla in studies using different classification systems, showing a higher tendency for post-ERCP pancreatitis in type II papilla, compared to the other papilla types

**Supplementary Figure 7.** Forest plot representing the influential analysis with the leave-one-out method in the outcome post-ERCP pancreatitis in studies using the Haraldsson classification

**Supplementary Figure 8.** Forest plot representing the influential analysis with the leave-one-out method in the outcome post-ERCP pancreatitis in studies using different classification systems

**Supplementary Figure 9.** Forest plot representing the pooled event rate of post-ERCP bleeding in the different papilla in studies using different classification systems, showing no statistically significant difference in the event rate between the papilla types

**Supplementary Figure 10.** Forest plot representing the influential analysis with the leave-one-out method in the outcome post-ERCP bleeding in studies using the Haraldsson classification

**Supplementary Figure 11.** Forest plot representing the influential analysis with the leave-one-out method in the outcome post-ERCP bleeding in studies using different classification systems

**Supplementary Figure 12.** Risk of bias assessment on study level [A] and across studies [B] for difficult cannulation

**Supplementary Figure 13.** Risk of bias assessment on study level [A] and across studies [B] for cannulation failure

**Supplementary Figure 14.** Risk of bias assessment on study level [A] and across studies [B] for cannulation time

**Supplementary Figure 15.** Risk of bias assessment on study level [A] and across studies [B] for the number of cannulation attempts

**Supplementary Figure 16.** Risk of bias assessment on study level [A] and across studies [B] for post-ERCP pancreatitis

**Supplementary Figure 17.** Risk of bias assessment on study level [A] and across studies [B] for post-ERCP bleeding

**Supplementary Figure 18.** Risk of bias assessment on study level [A] and across studies [B] for post-ERCP perforation

**Supplementary Figure 19.** Risk of bias assessment on study level [A] and across studies [B] for post-ERCP infection

**Supplementary Figure 20.** Funnel plot representing publication bias assessed with Egger's test in the outcome difficult cannulation in studies using the Haraldsson classification

**Supplementary Figure 21.** Funnel plot representing publication bias assessed with Egger's test, in the outcome difficult cannulation in studies using different classification systems

**Supplementary Figure 22.** Funnel plot representing publication bias assessed with Egger's test in the outcome cannulation failure in studies using the Haraldsson classification

**Supplementary Figure 23.** Funnel plot representing publication bias assessed with Egger's test in the outcome cannulation failure in studies using different classification systems

**Supplementary Figure 24.** Funnel plot representing publication bias assessed with Egger's test in the outcome post-ERCP pancreatitis in studies using the Haraldsson classification

**Supplementary Figure 25.** Funnel plot representing publication bias assessed with Egger's test in the outcome post-ERCP pancreatitis in studies using different classification systems

**Supplementary Figure 26.** Funnel plot representing publication bias assessed with Egger's test in the outcome post-ERCP bleeding in studies using the Haraldsson classification

**Supplementary Figure 27.** Funnel plot representing publication bias assessed with Egger's test in the outcome post-ERCP bleeding in studies using different classification systems

**TABLE LEGENDS**

**Supplementary Table 1.** Detailed search strategy

**Supplementary Table 2.** Systematic review table for outcomes detailing the cannulation process

**Supplementary Table 3.** Systematic review table for outcomes detailing the post-ERCP adverse events

**Supplementary Table 4.** Summary of findings table for difficult cannulation in studies using the Haraldsson classification

**Supplementary Table 5.** Summary of findings table for difficult cannulation in studies using different classifications

**Supplementary Table 6.** Summary of findings table for cannulation failure in studies using the Haraldsson classification

**Supplementary Table 7.** Summary of findings table for cannulation failure in studies using different classifications

**Supplementary Table 8.** Summary of findings table for post-ERCP pancreatitis in studies using the Haraldsson classification

**Supplementary Table 9.** Summary of findings table for post-ERCP pancreatitis in studies using different classifications

**Supplementary Table 10.** Summary of findings table for post-ERCP bleeding in studies using the Haraldsson classification

**Supplementary Table 11.** Summary of findings table for post-ERCP bleeding in studies using different classifications

**Supplementary Table 12**. PRISMA 2020 checklist

**ADDITIONAL DETAILS ON STATISTICAL ANALYSES**


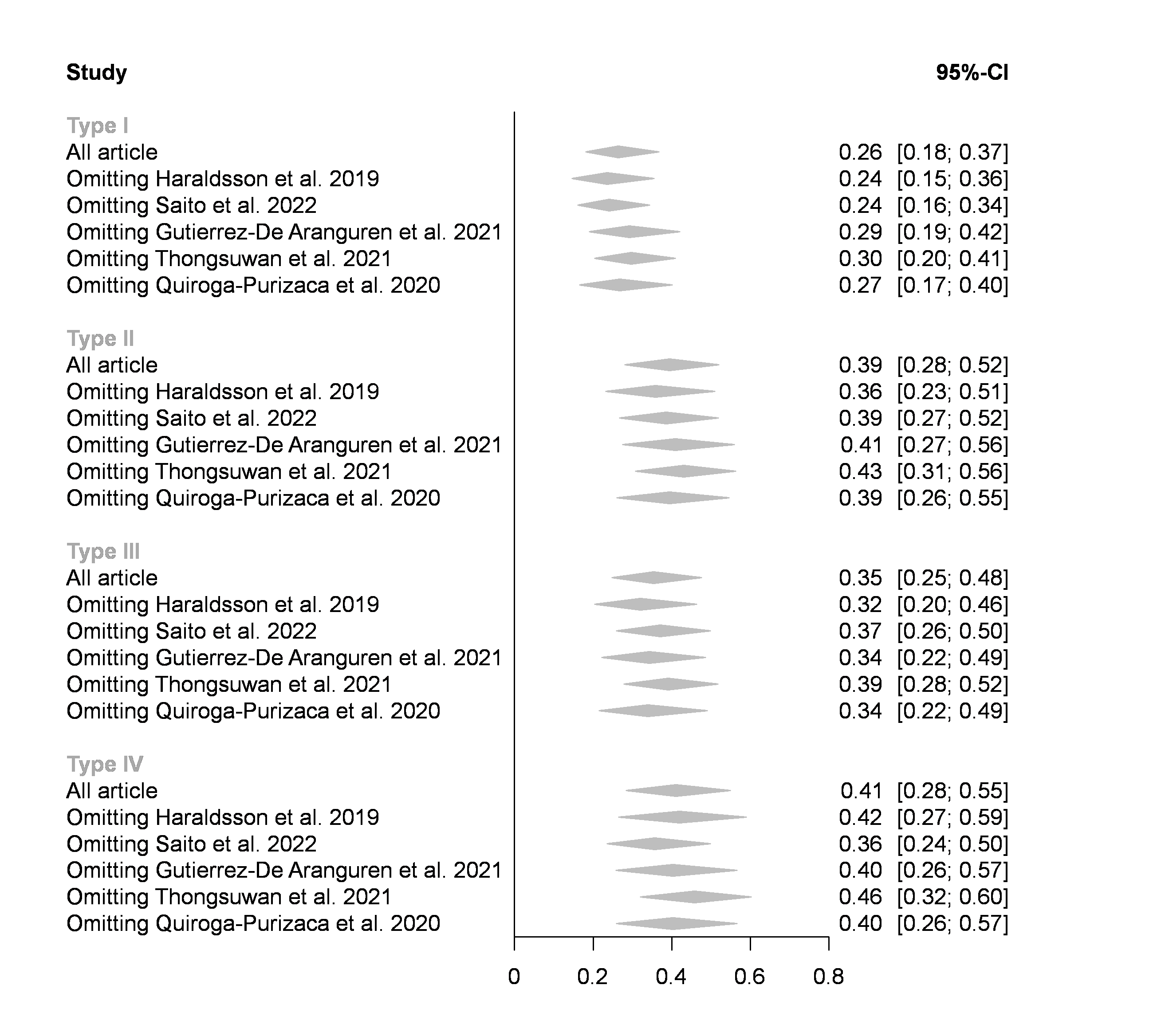


**Supplementary Figure 1.** Forest plot representing the influential analysis with the leave-one-out method in the outcome of difficult cannulation in studies using the Haraldsson classification


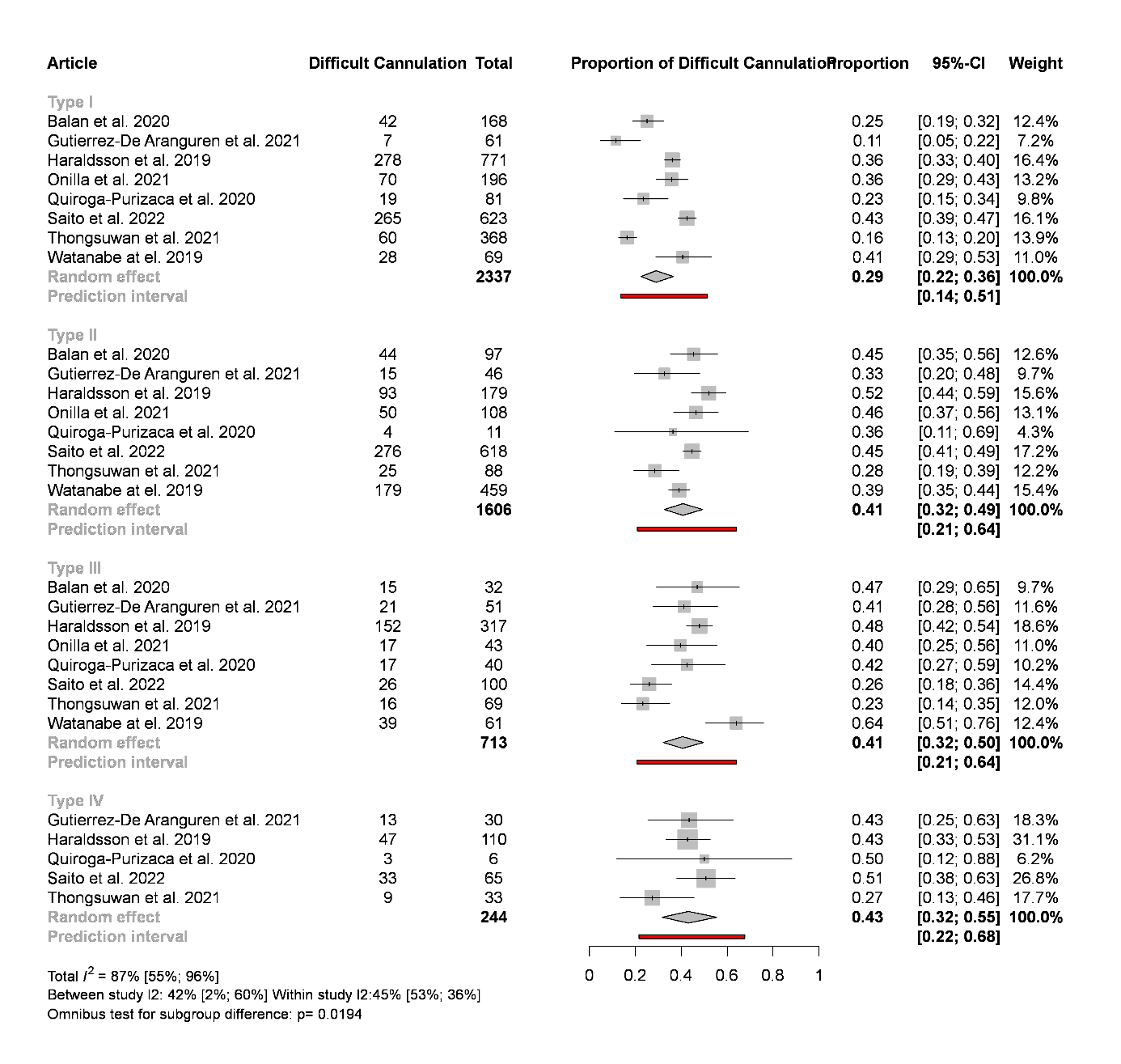


**Supplementary Figure 2.** Forest plot representing the pooled event rate of difficult cannulation in the different papilla types in studies using different classification systems, showing statistically significantly lower rate in type I papilla, compared to the other papilla types


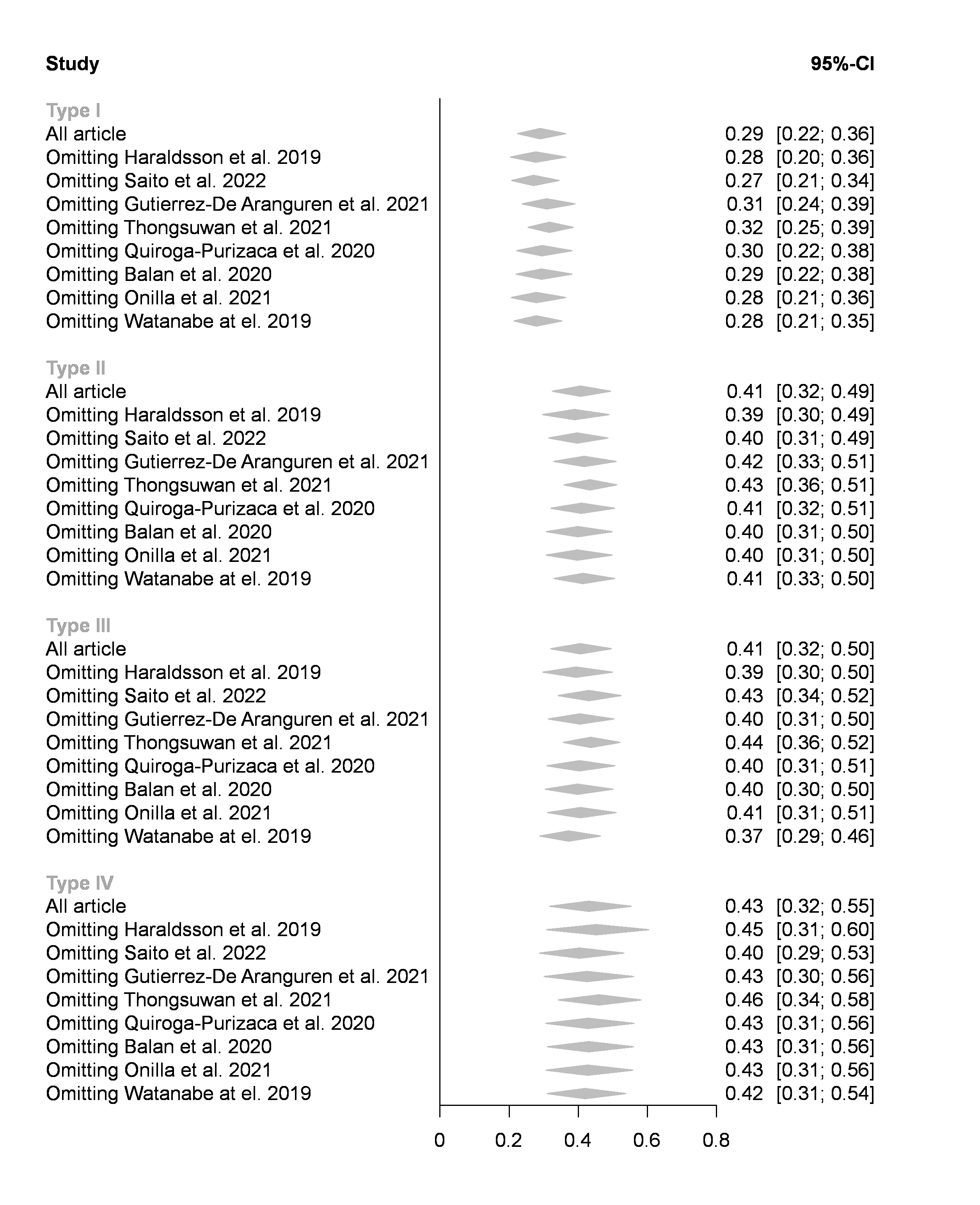


**Supplementary Figure 3.** Forest plot representing the influential analysis with the leave-one-out method in the outcome of difficult cannulation in studies using different classification systems


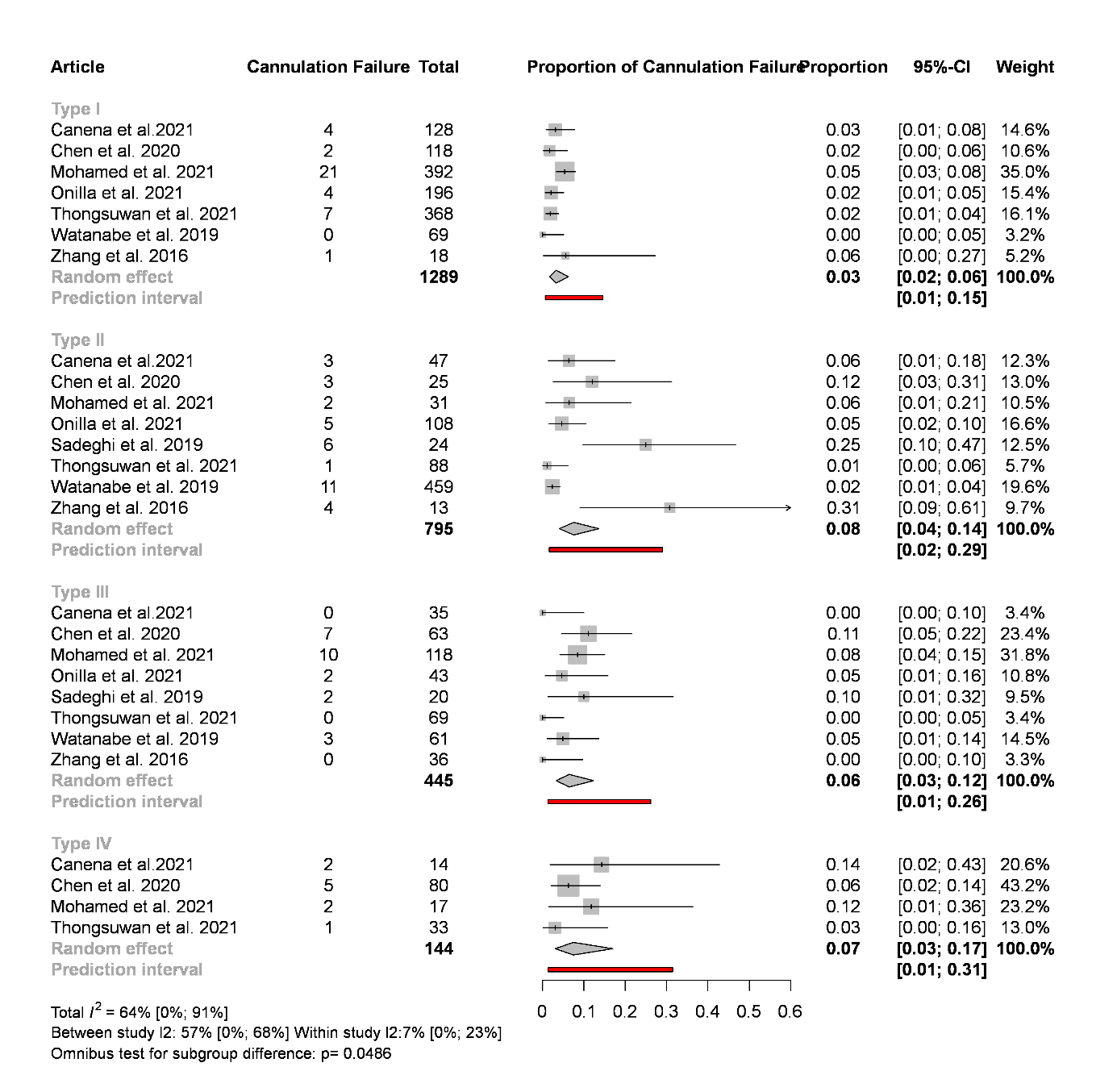


**Supplementary Figure 4.** Forest plot representing the pooled event rate of cannulation failure in the different papilla types in studies using different classification systems, showing statistically significant difference in the event rates between the papilla types


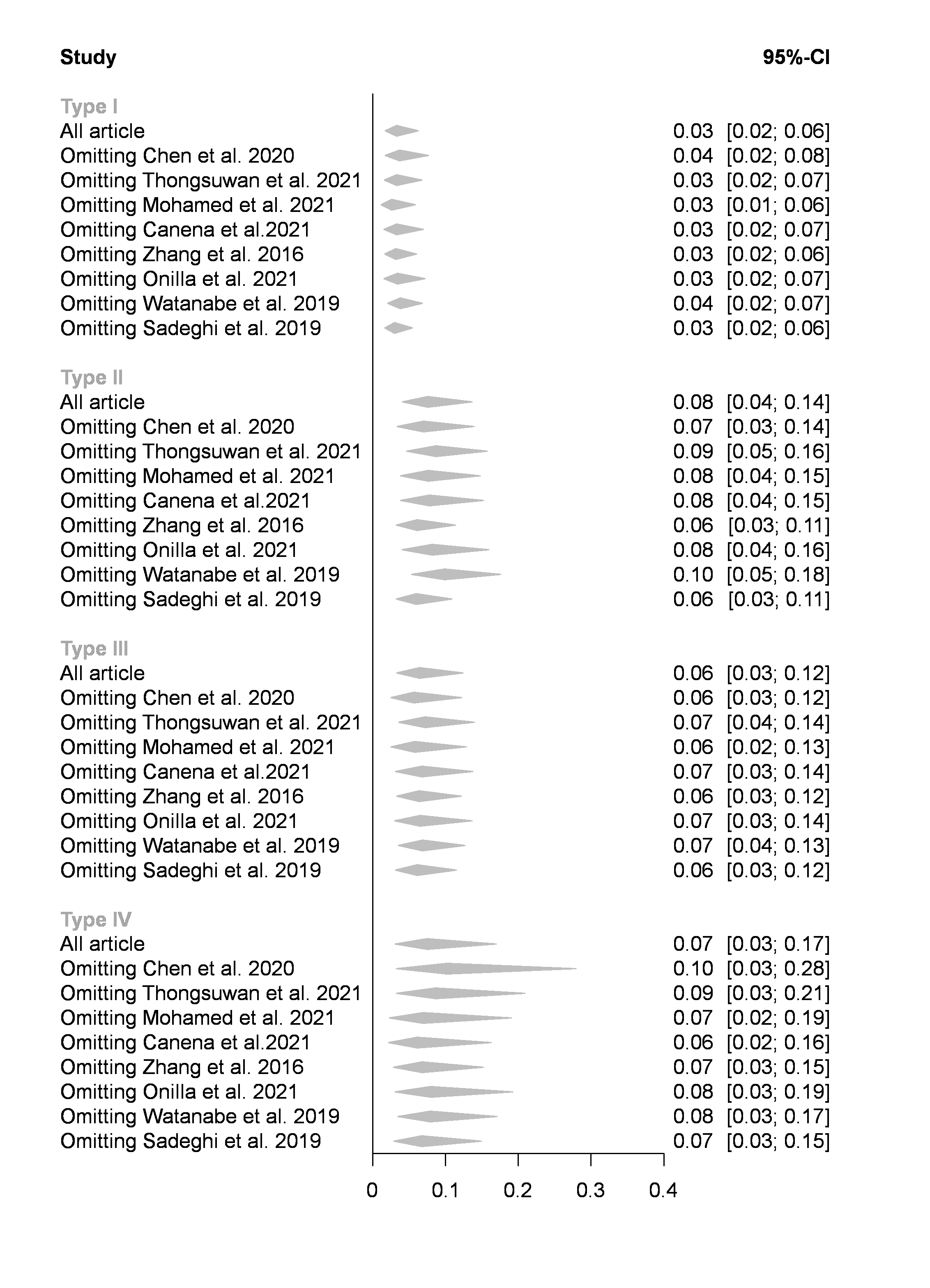


**Supplementary Figure 5.** Forest plot representing the influential analysis with the leave-one-out method in the outcome cannulation failure in studies using different classification systems


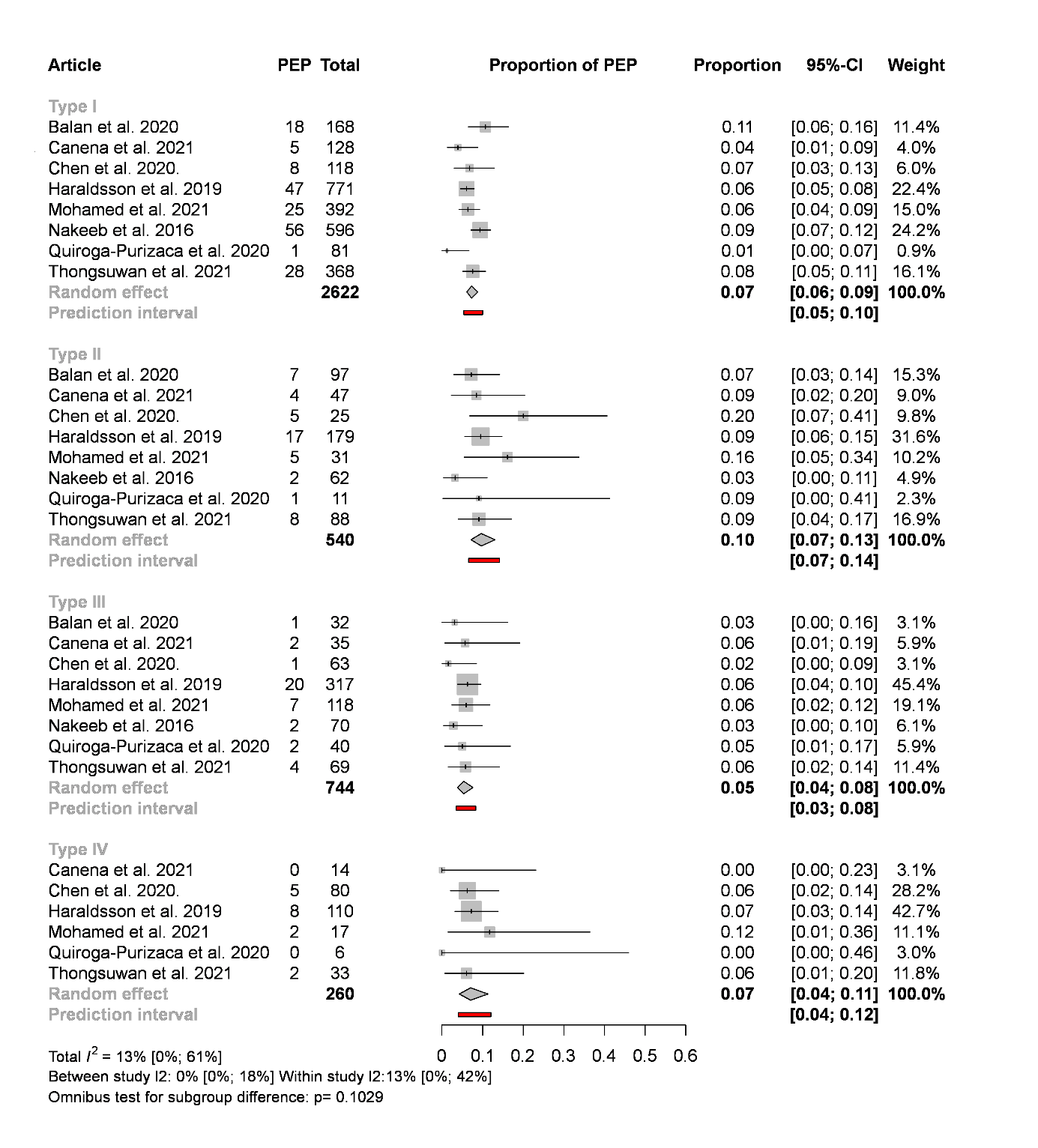


**Supplementary Figure 6.** Forest plot representing the pooled event rate of post-ERCP pancreatitis in the different papilla in studies using different classification systems, showing a higher tendency for post-ERCP pancreatitis in type II papilla, compared to the other papilla types


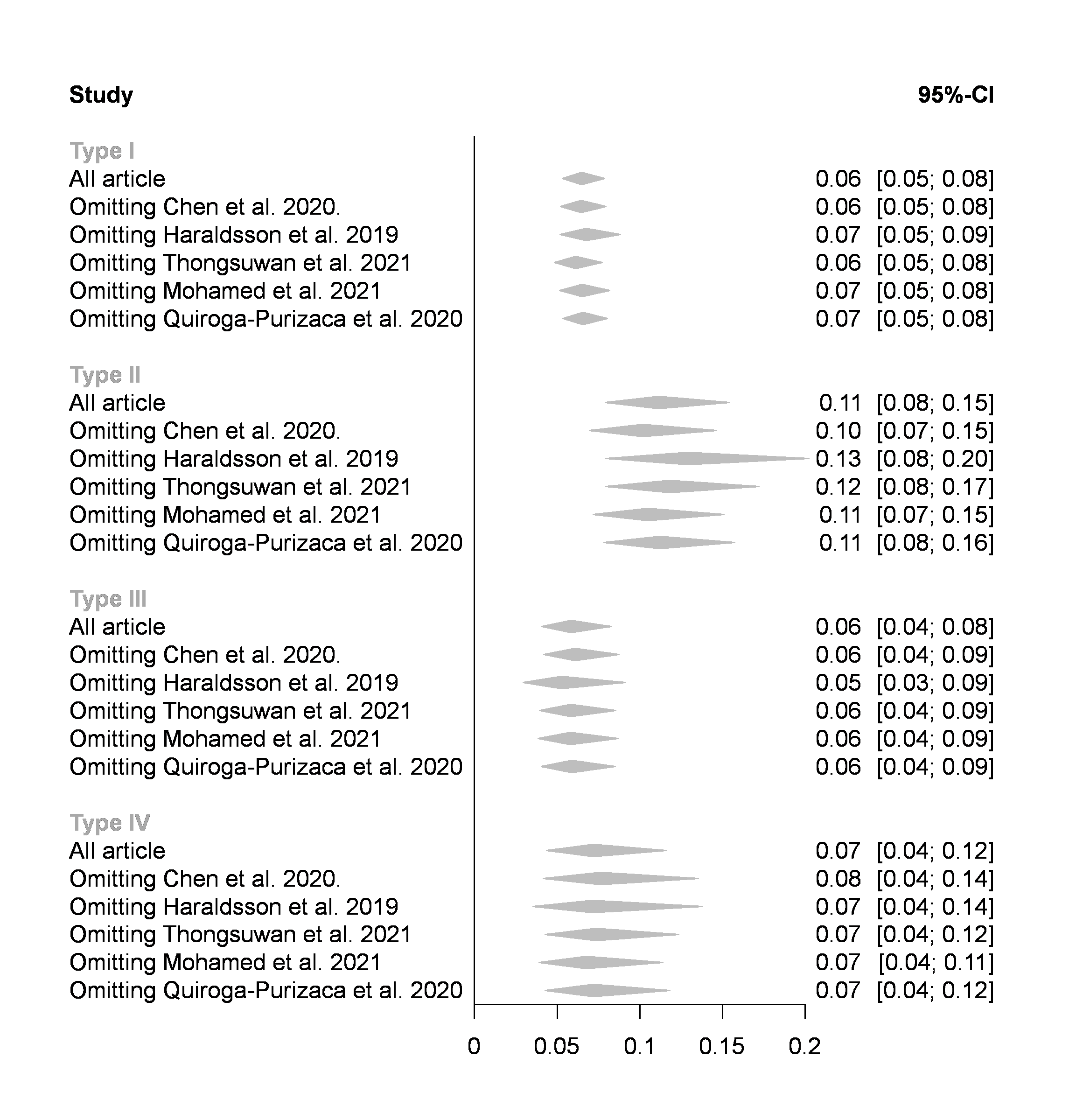


**Supplementary Figure 7.** Forest plot representing the influential analysis with the leave-one-out method in the outcome post-ERCP pancreatitis in studies using the Haraldsson classification


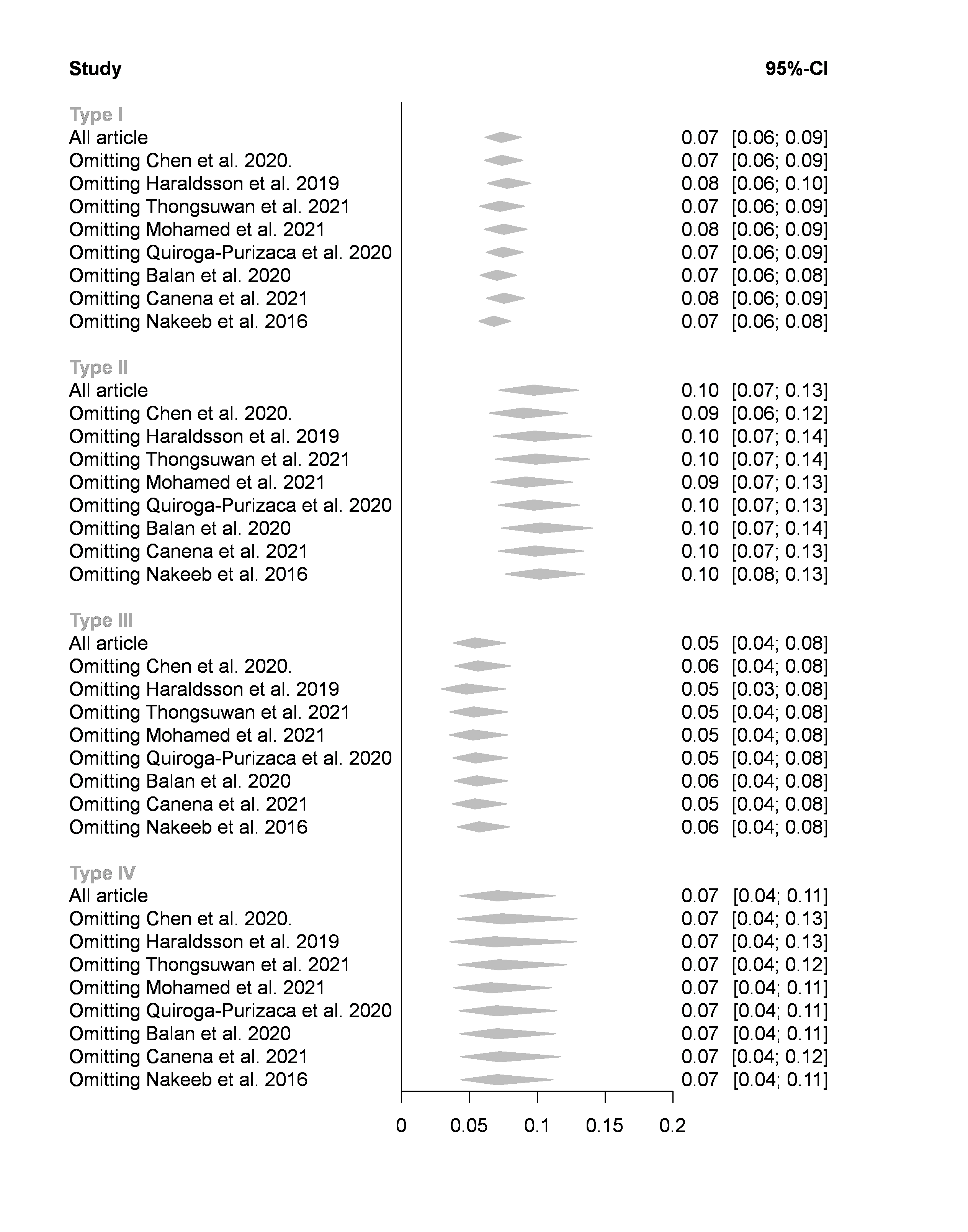


**Supplementary Figure 8.** Forest plot representing the influential analysis with the leave-one-out method in the outcome post-ERCP pancreatitis in studies using different classification systems


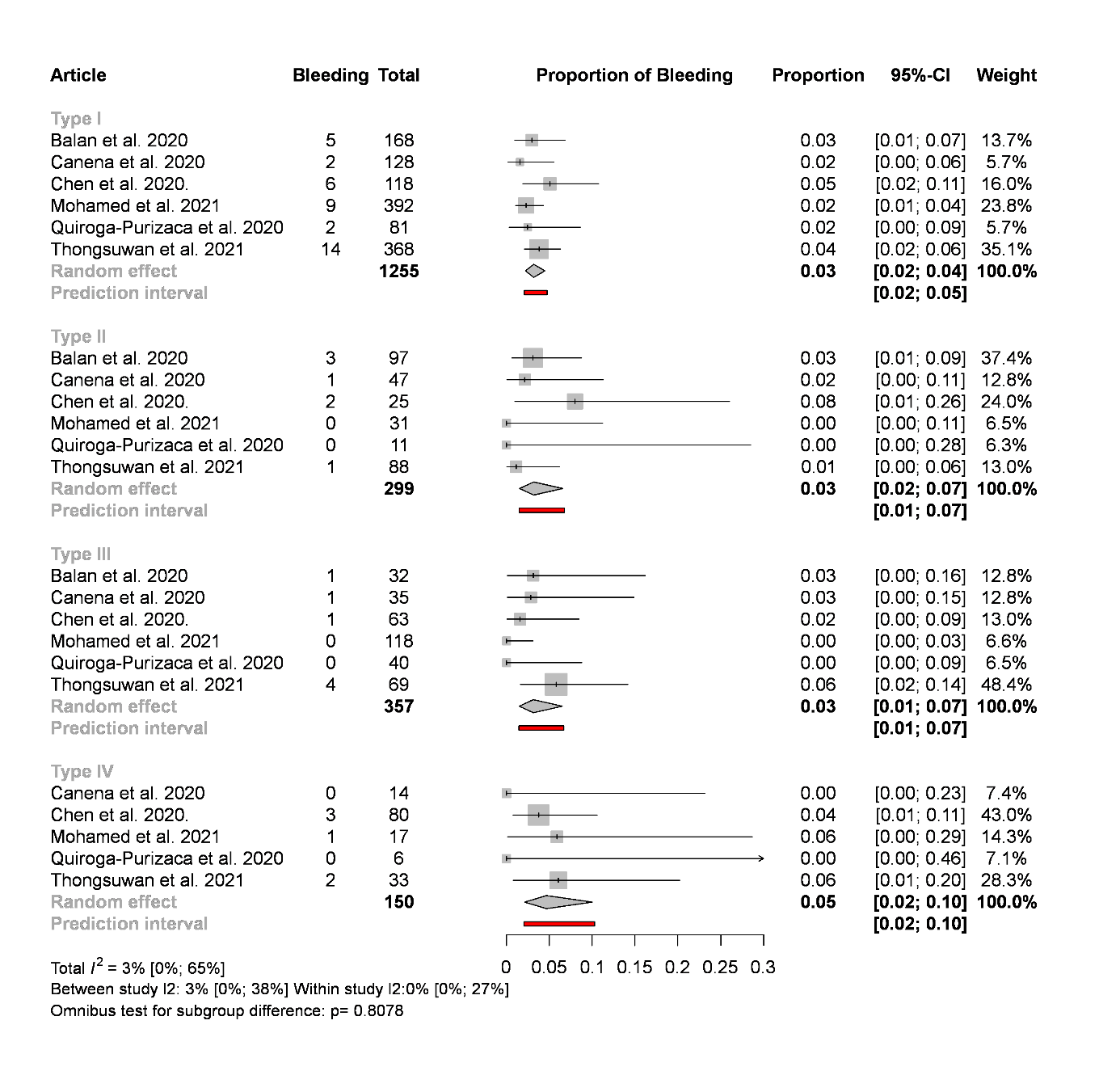


**Supplementary Figure 9.** Forest plot representing the pooled event rate of post-ERCP bleeding in the different papilla in studies using different classification systems, showing no statistically significant difference in the event rate between the papilla types


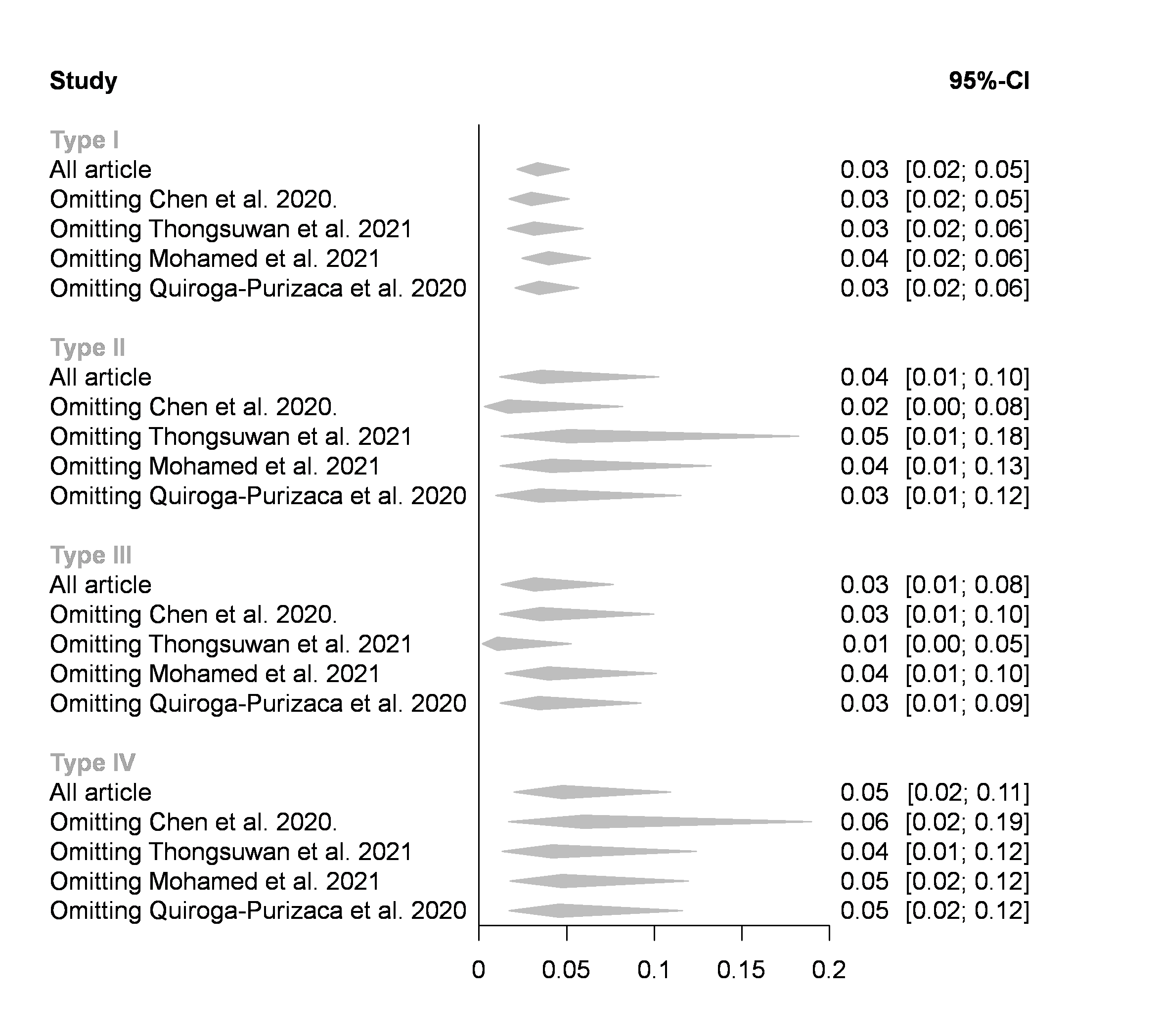


**Supplementary Figure 10.** Forest plot representing the influential analysis with the leave-one-out method in the outcome post-ERCP bleeding in studies using the Haraldsson classification


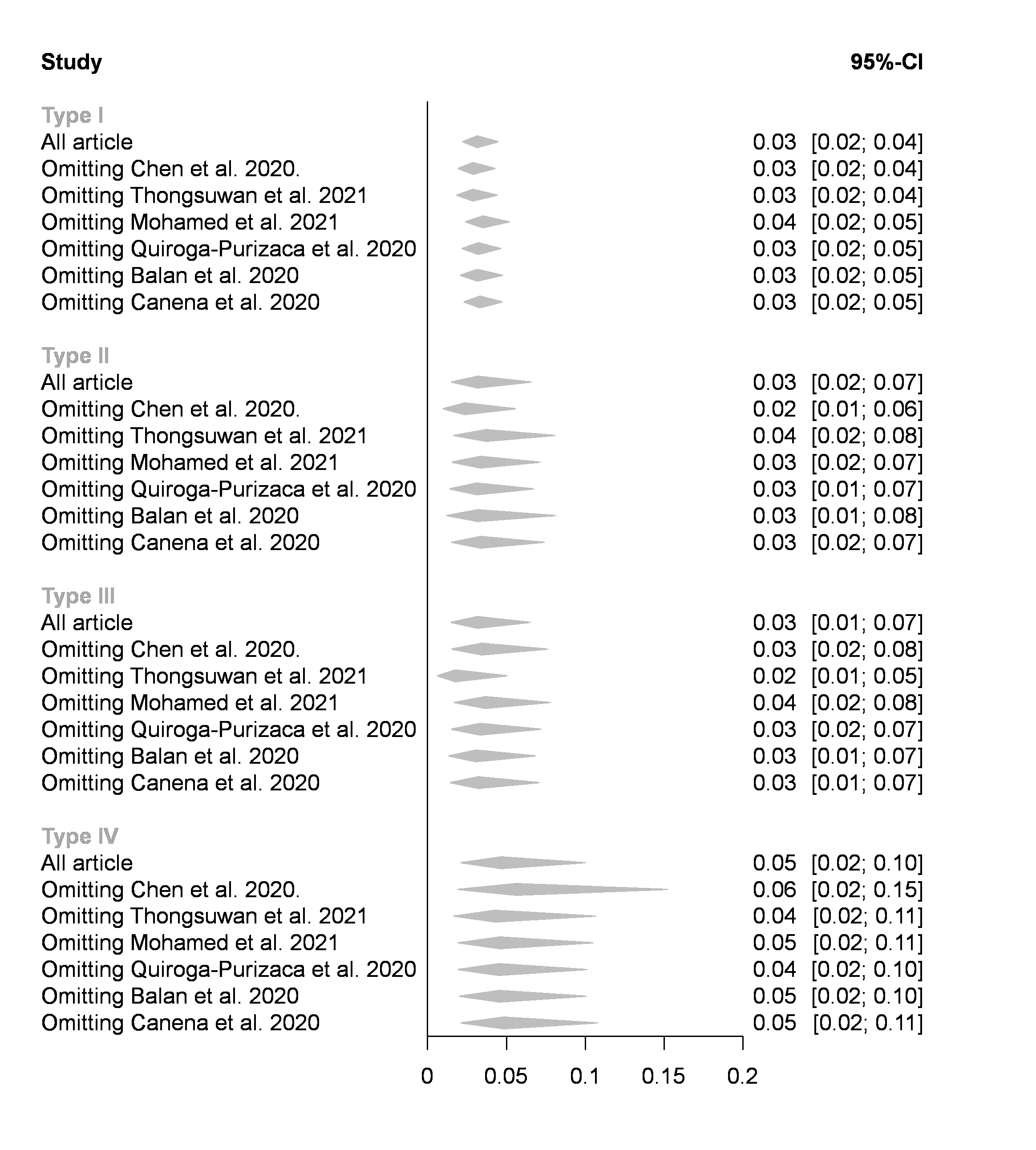


**Supplementary Figure 11.** Forest plot representing the influential analysis with the leave-one-out method in the outcome post-ERCP bleeding in studies using different classification systems


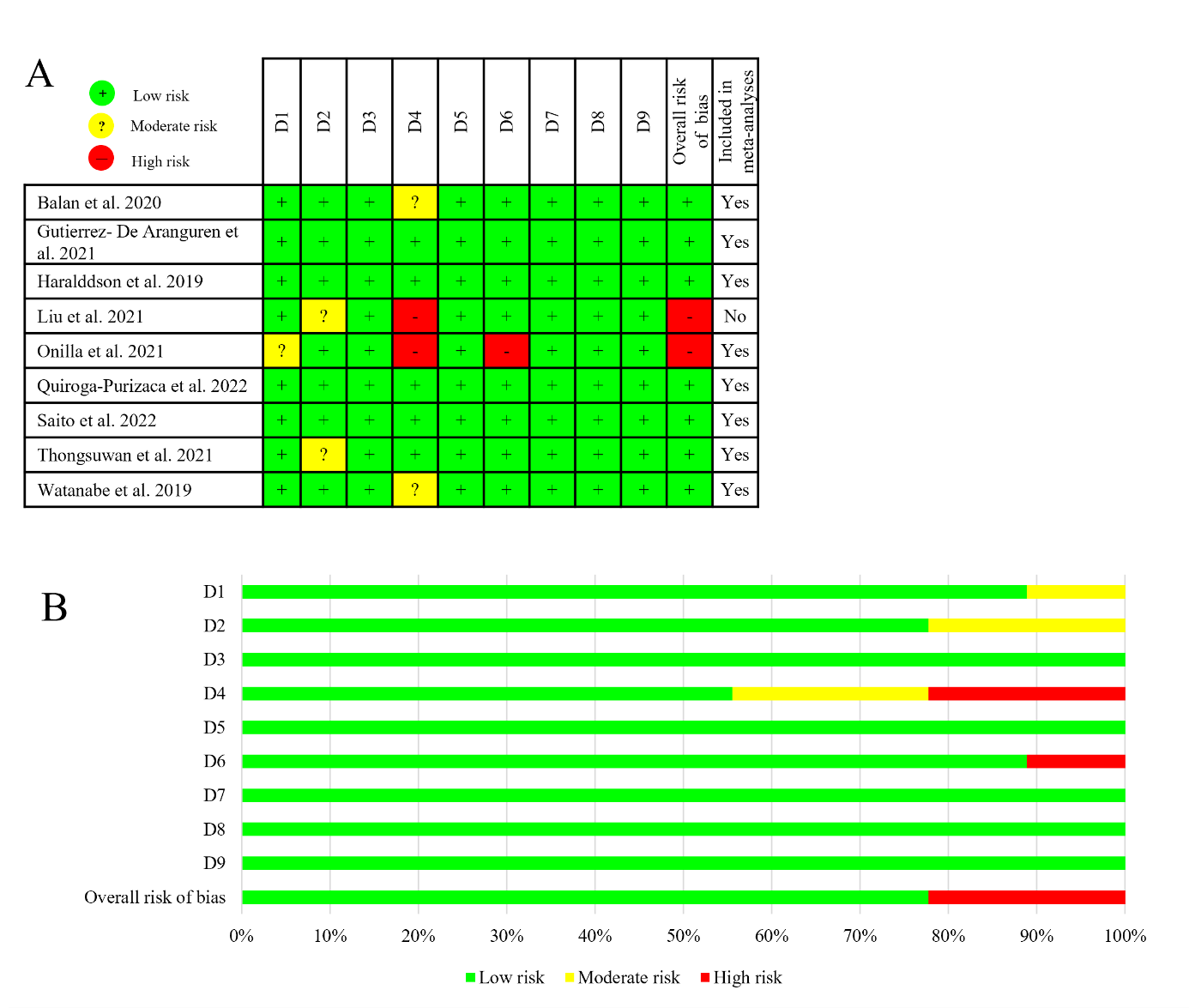


**Supplementary Figure 12.** Risk of bias assessment on study level [A] and across studies [B] for difficult cannulation


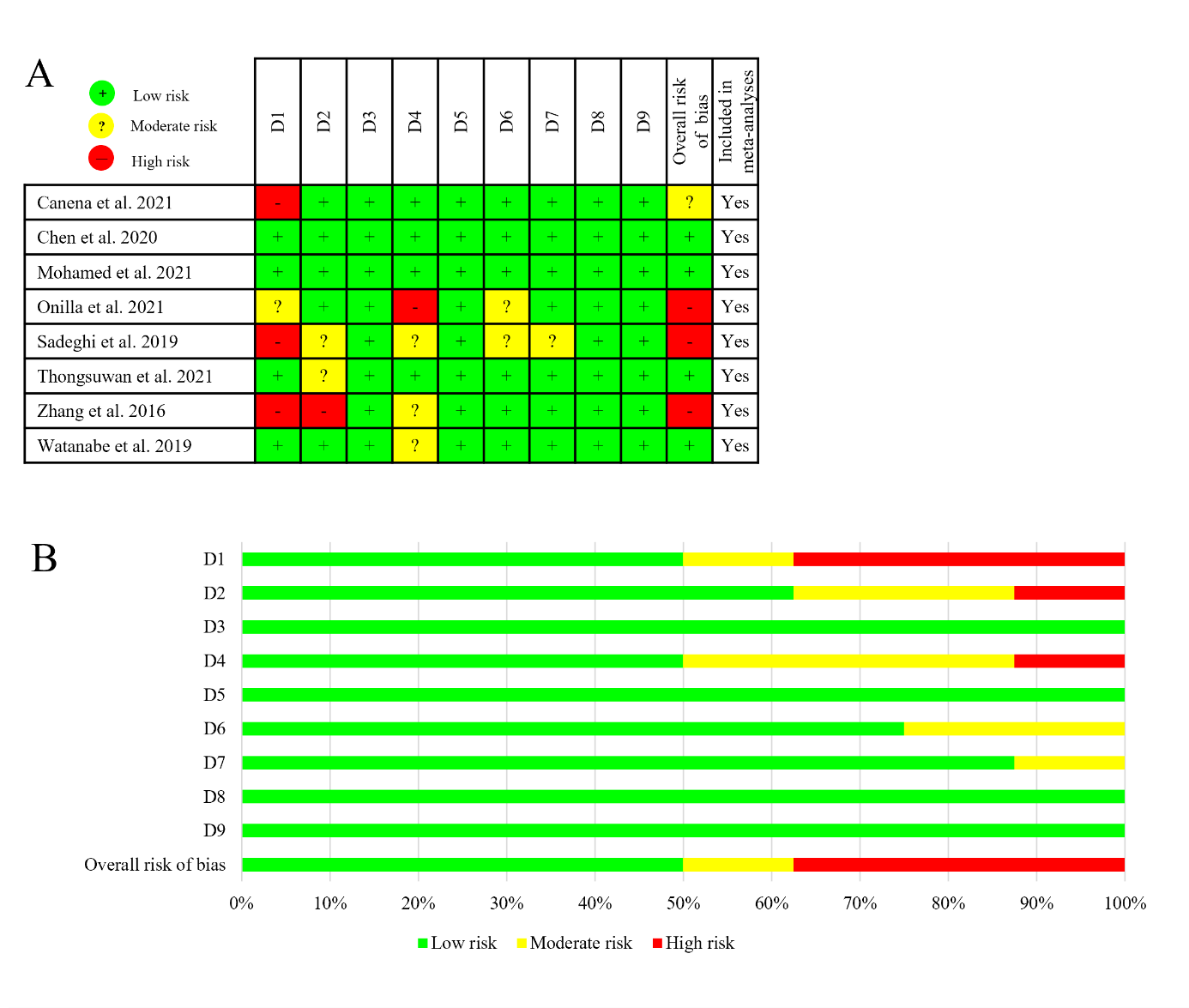


**Supplementary Figure 13.** Risk of bias assessment on study level [A] and across studies [B] for cannulation failure


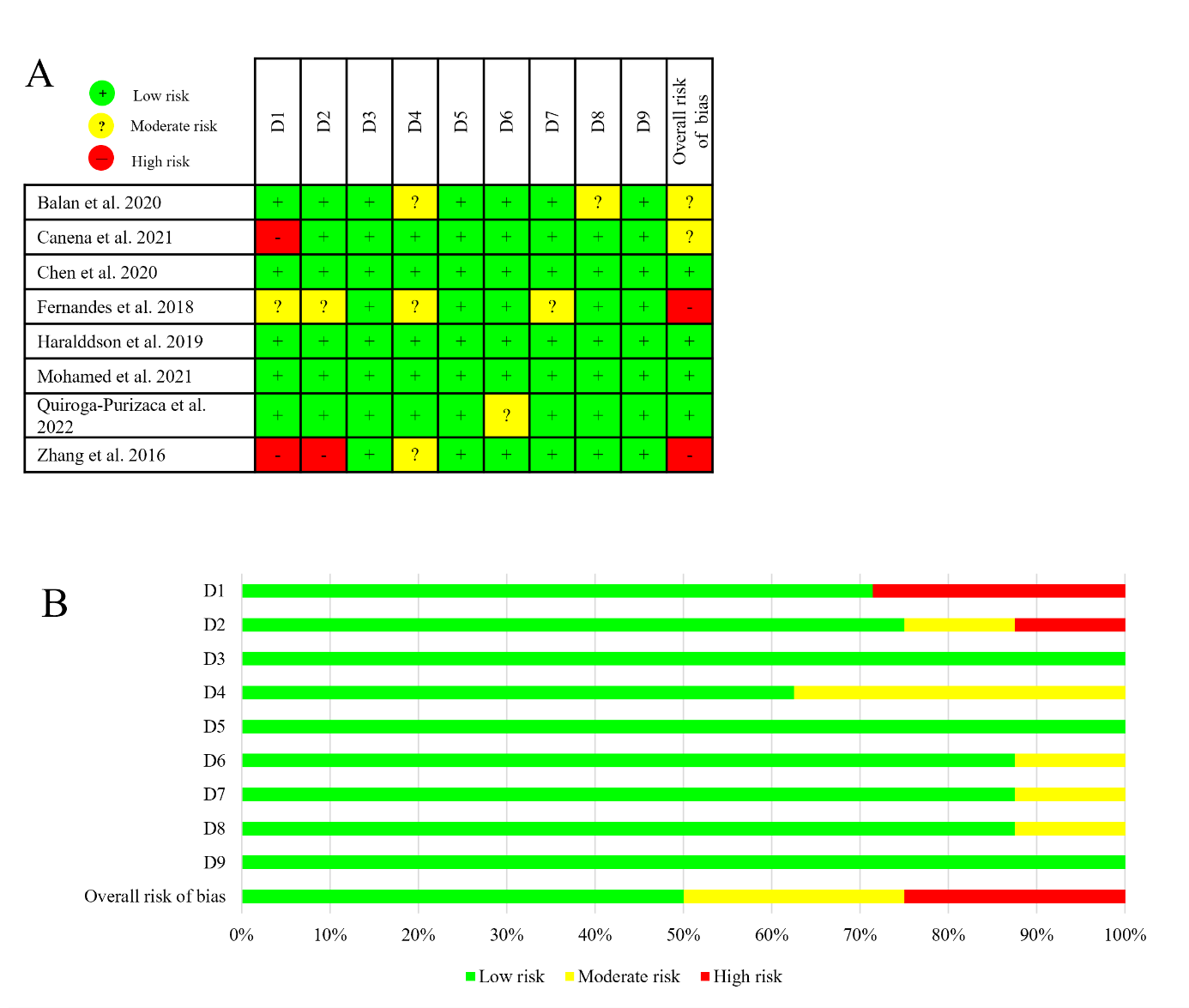


**Supplementary Figure 14.** Risk of bias assessment on study level [A] and across studies [B] for cannulation time


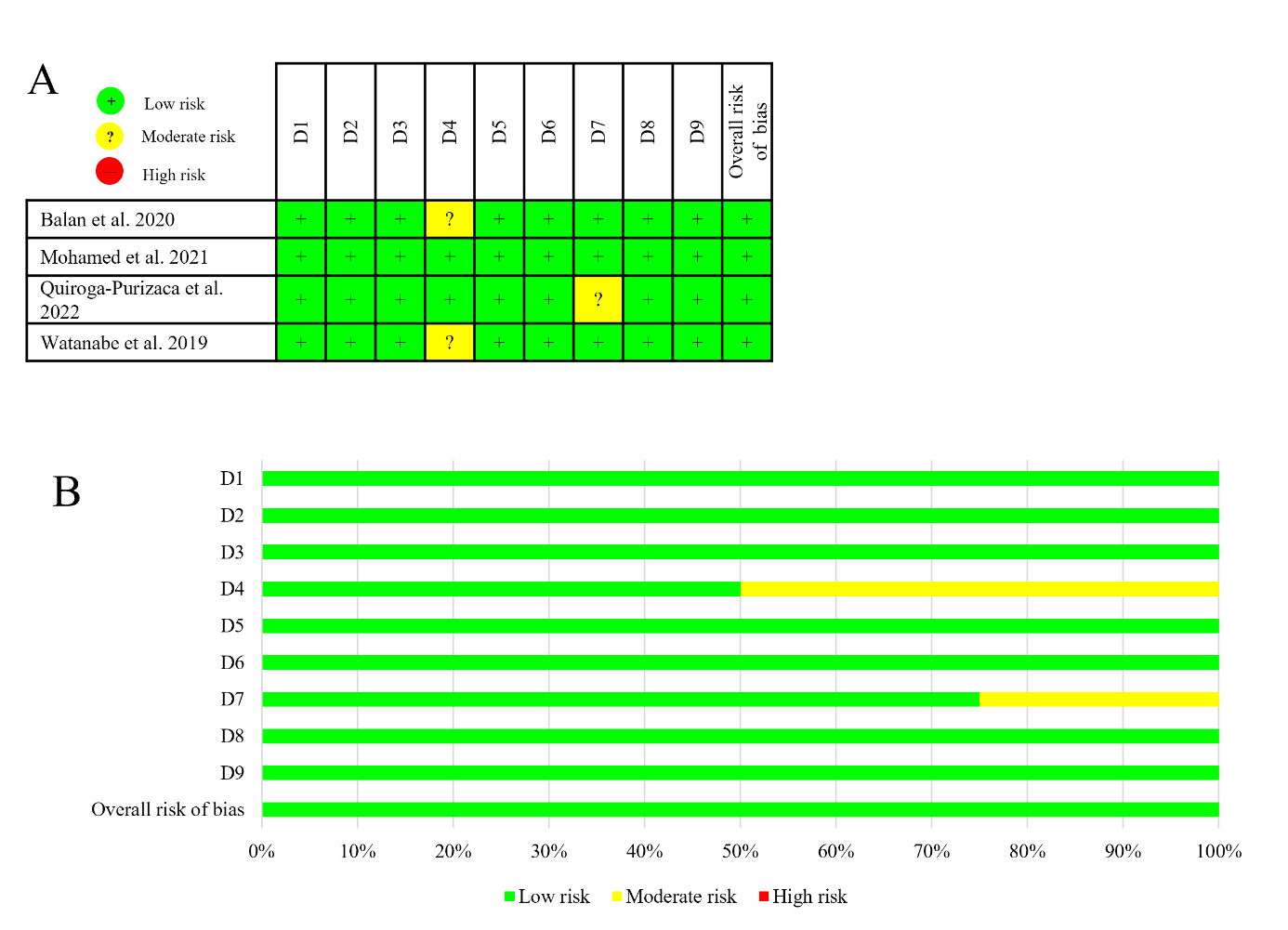


**Supplementary Figure 15.** Risk of bias assessment on study level [A] and across studies [B] for the number of cannulation attempts


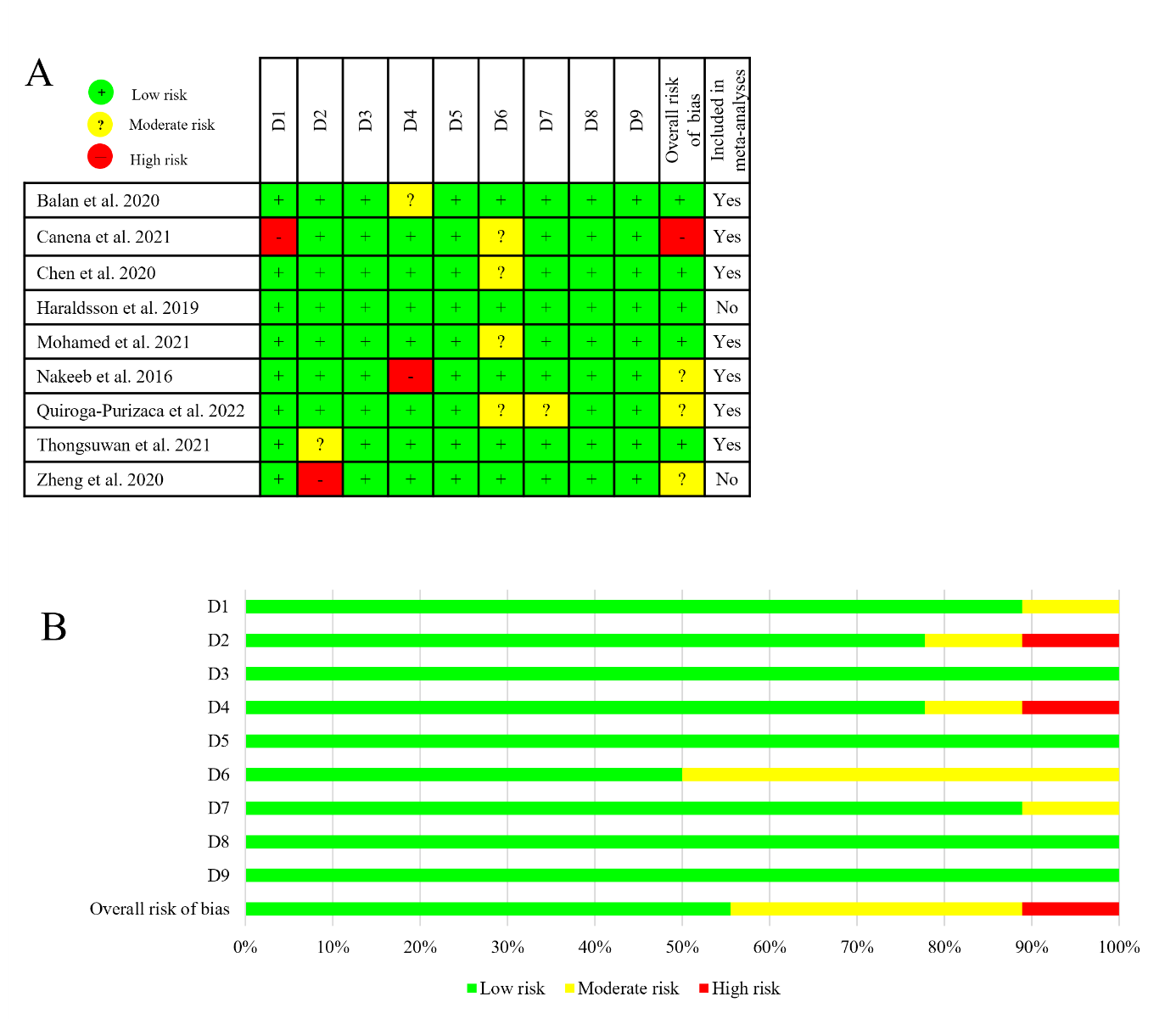


**Supplementary Figure 16.** Risk of bias assessment on study level [A] and across studies [B] for post-ERCP pancreatitis


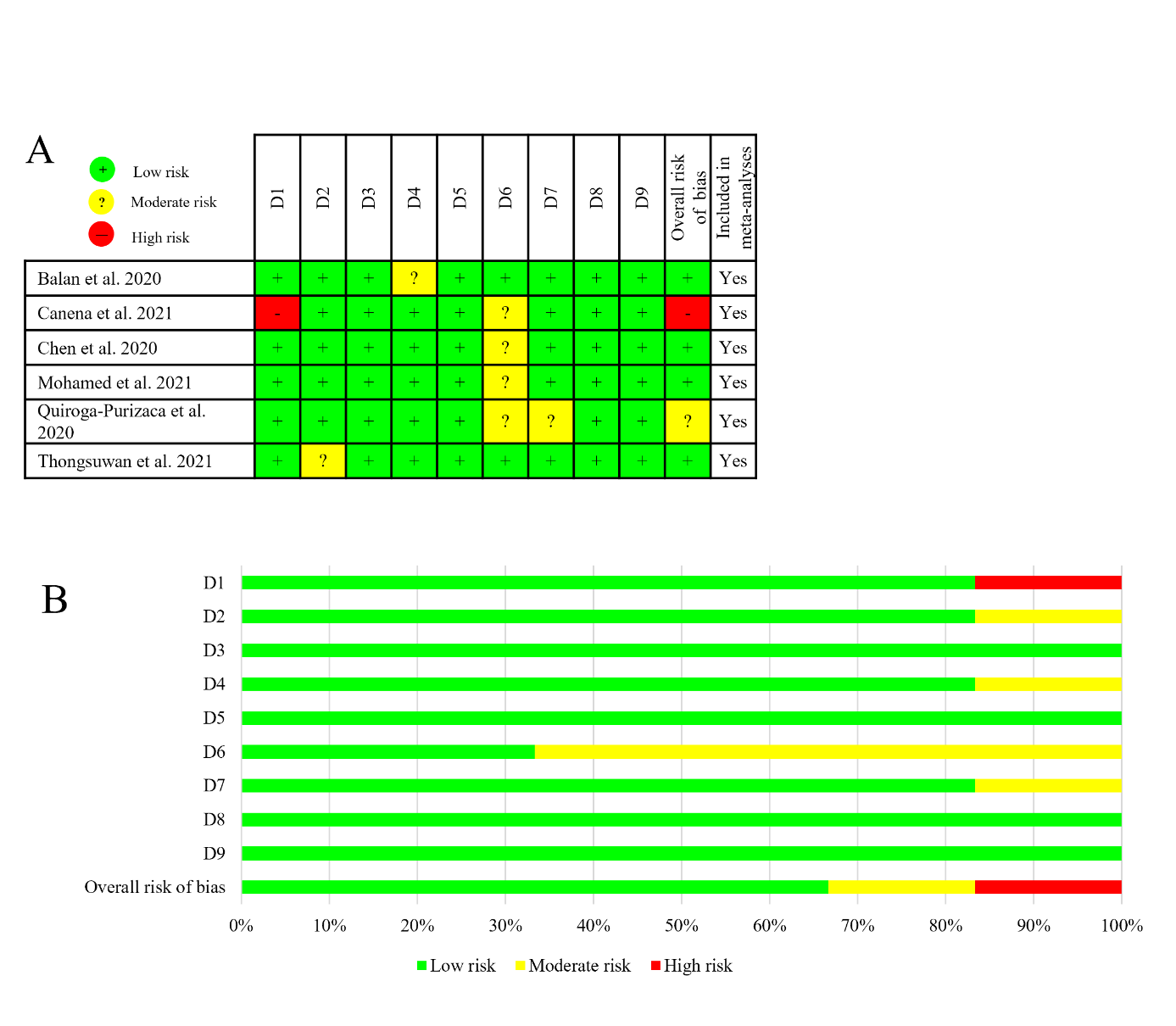


**Supplementary Figure 17.** Risk of bias assessment on study level [A] and across studies [B] for post-ERCP bleeding


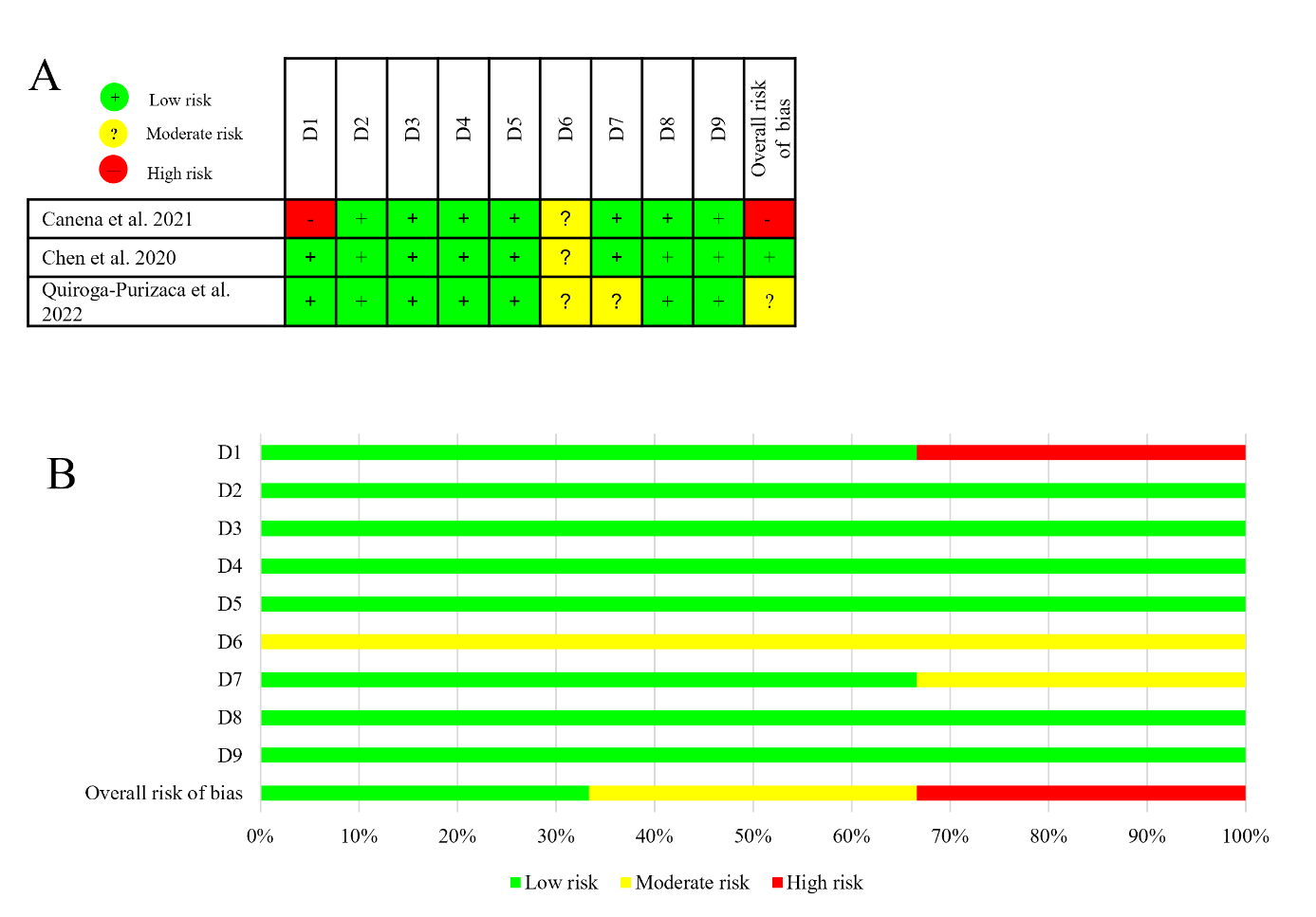


**Supplementary Figure 18.** Risk of bias assessment on study level [A] and across studies [B] for post-ERCP perforation


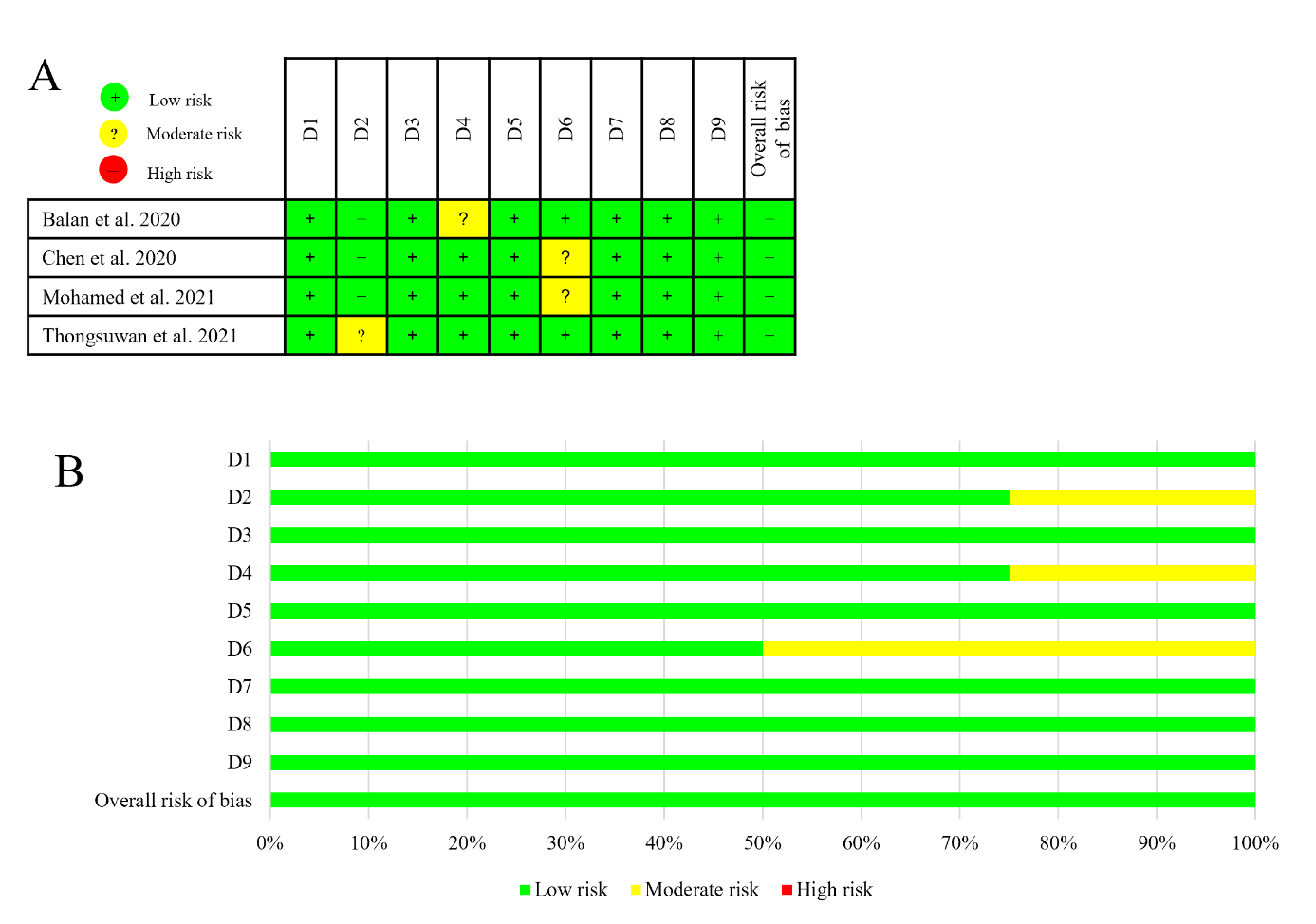


**Supplementary Figure 19.** Risk of bias assessment on study level [A] and across studies [B] for post-ERCP infection


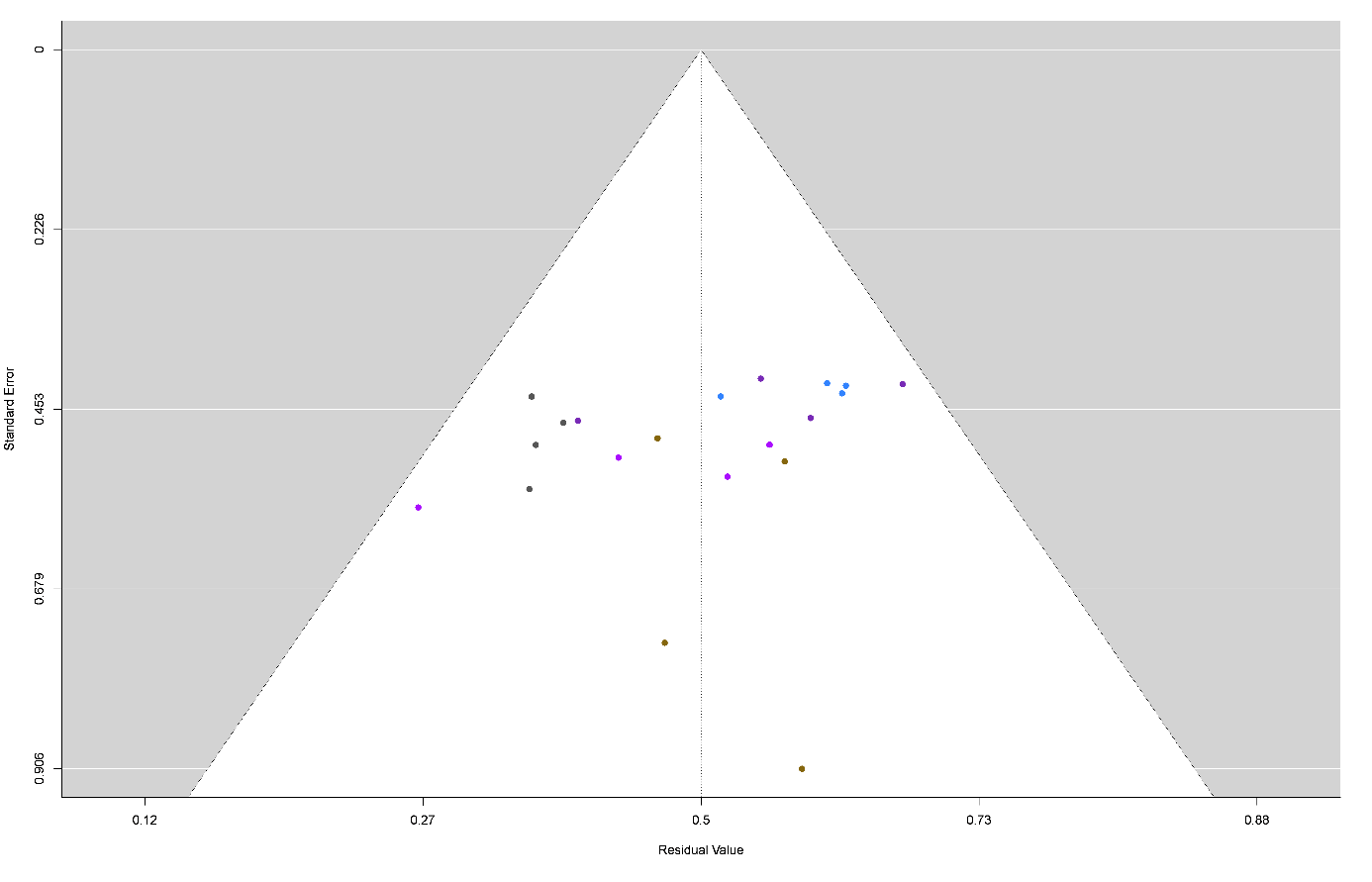


**Supplementary Figure 20.** Funnel plot representing publication bias assessed with Egger's test, in the outcome difficult cannulation in studies using the Haraldsson classification


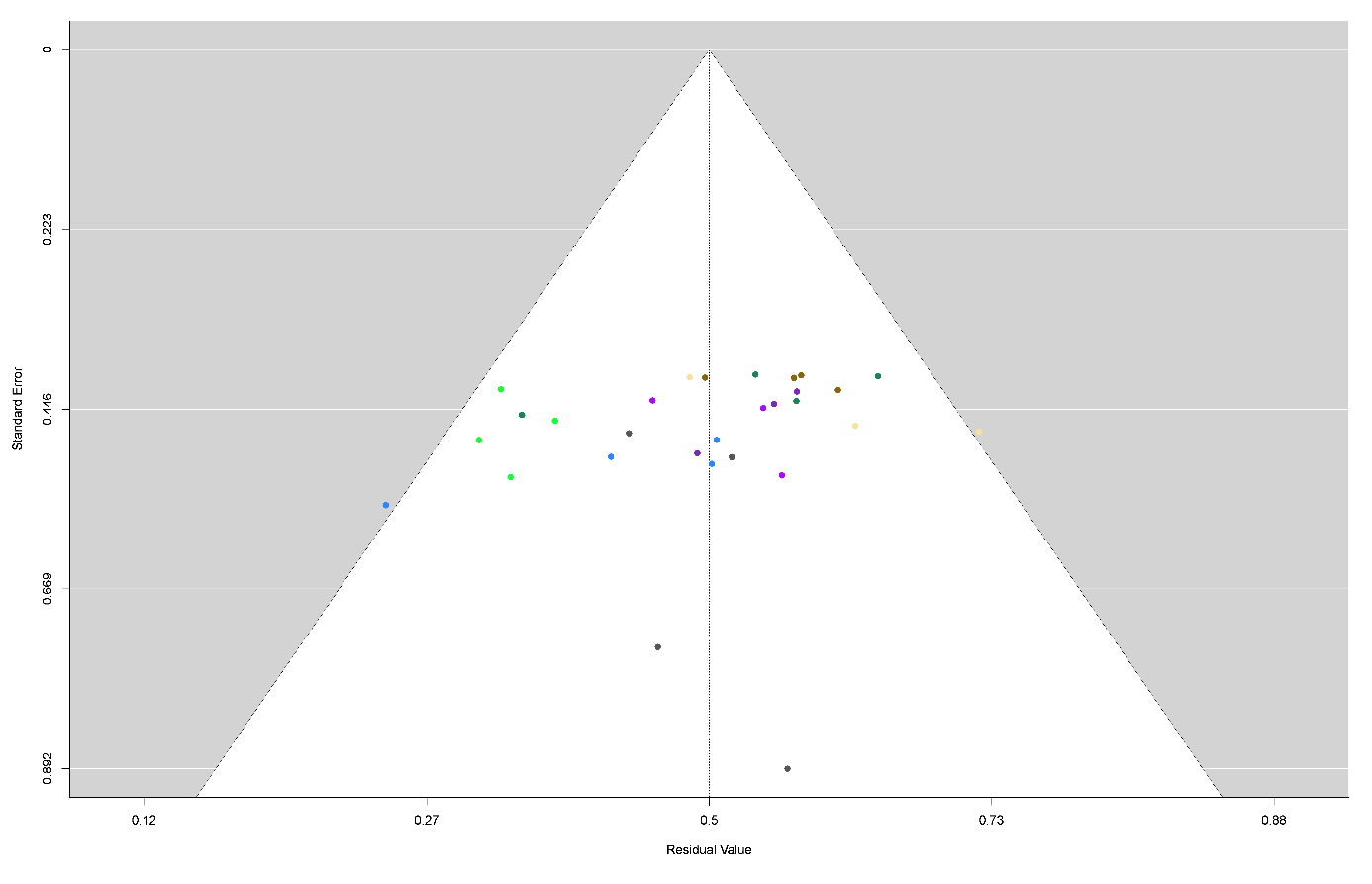


**Supplementary Figure 21.** Funnel plot representing publication bias assessed with Egger's test, in the outcome difficult cannulation in studies using different classification systems


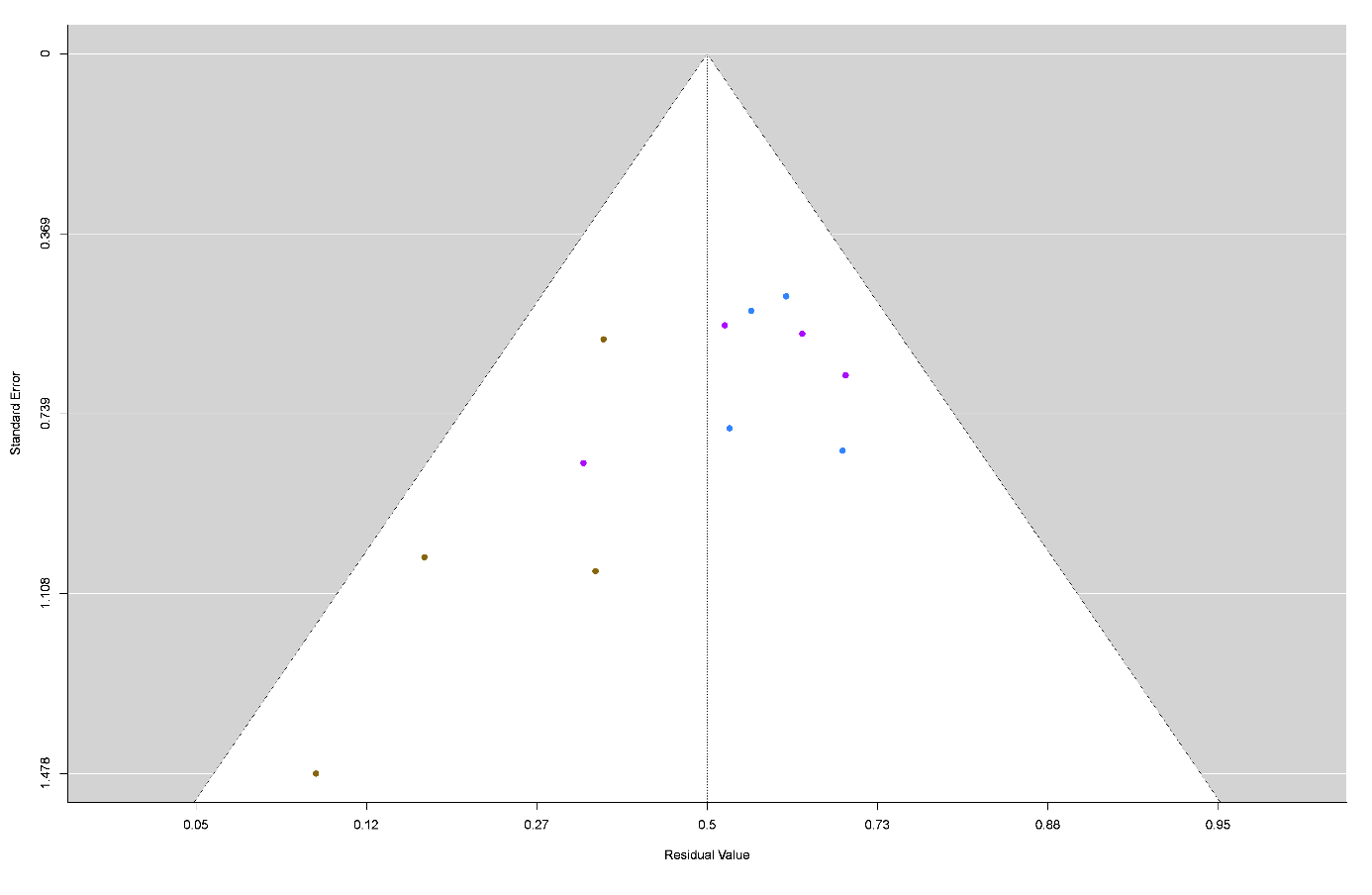


**Supplementary Figure 22.** Funnel plot representing publication bias assessed with Egger's test, in the outcome cannulation failure in studies using the Haraldsson classification


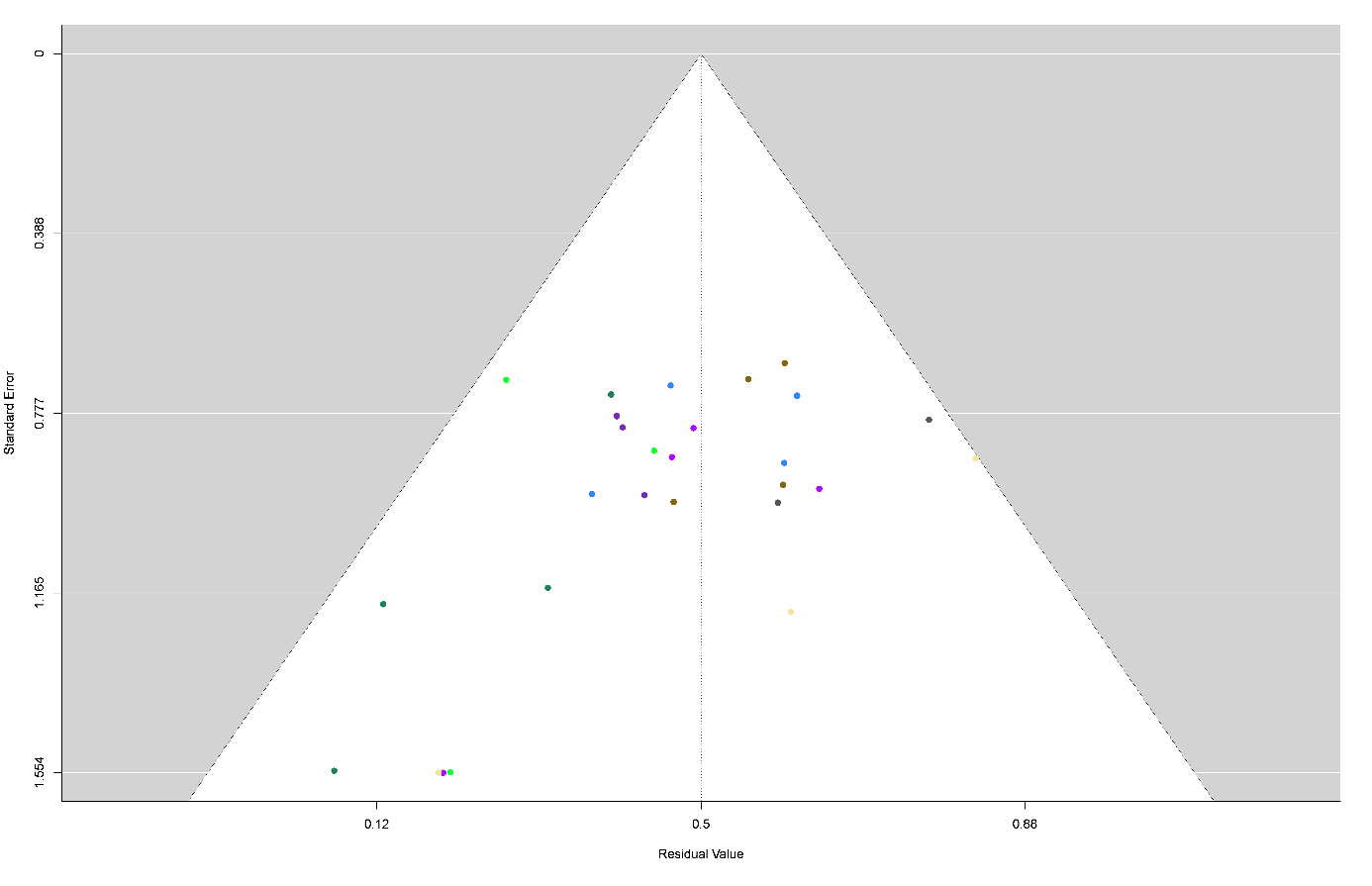


**Supplementary Figure 23.** Funnel plot representing publication bias assessed with Egger's test, in the outcome cannulation failure in studies using different classification systems


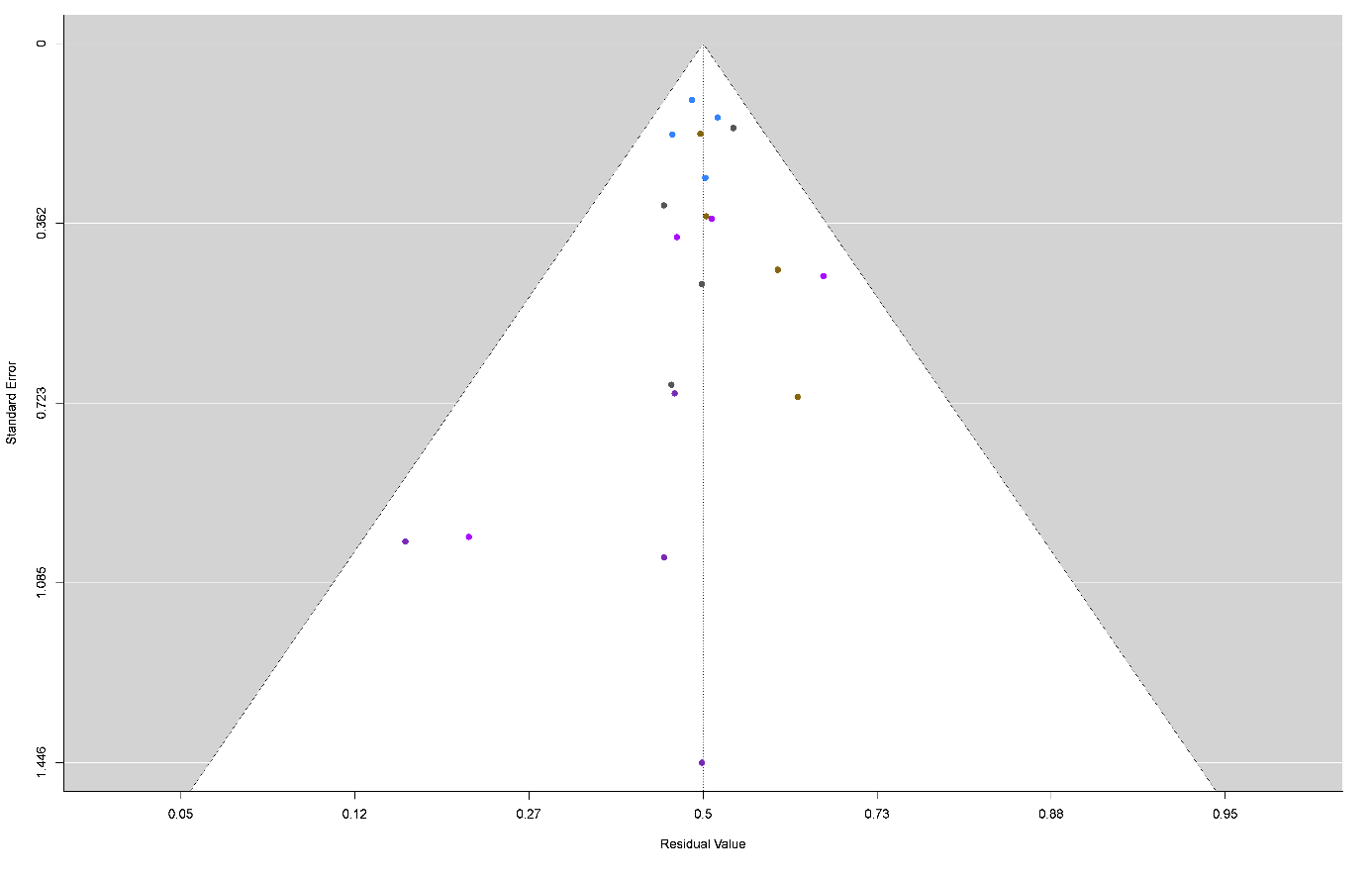


**Supplementary Figure 24.** Funnel plot representing publication bias assessed with Egger's test, in the outcome post-ERCP pancreatitis in studies using the Haraldsson classification


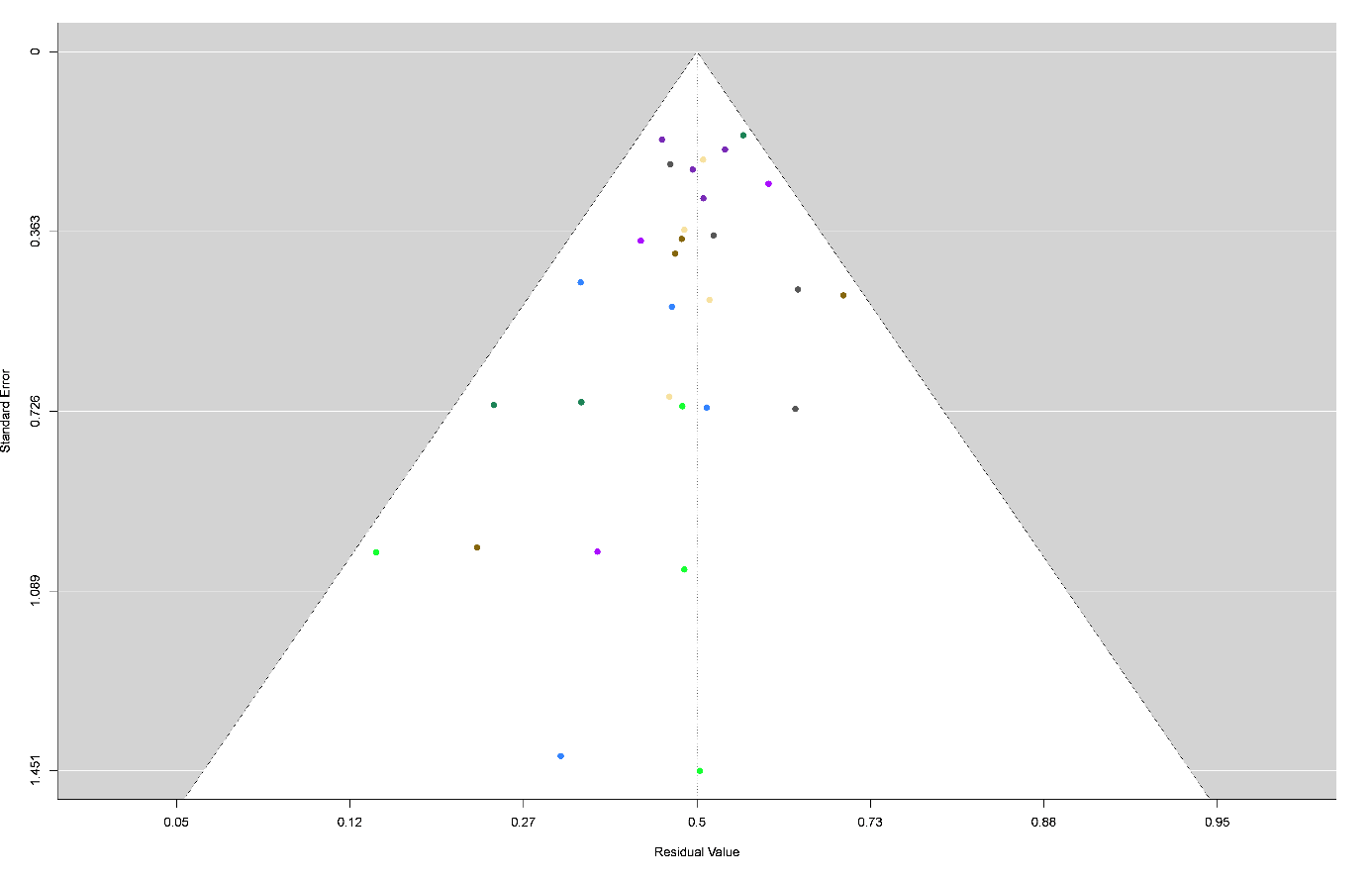


**Supplementary Figure 25.** Funnel plot representing publication bias assessed with Egger's test, in the outcome post-ERCP pancreatitis in studies using different classification systems


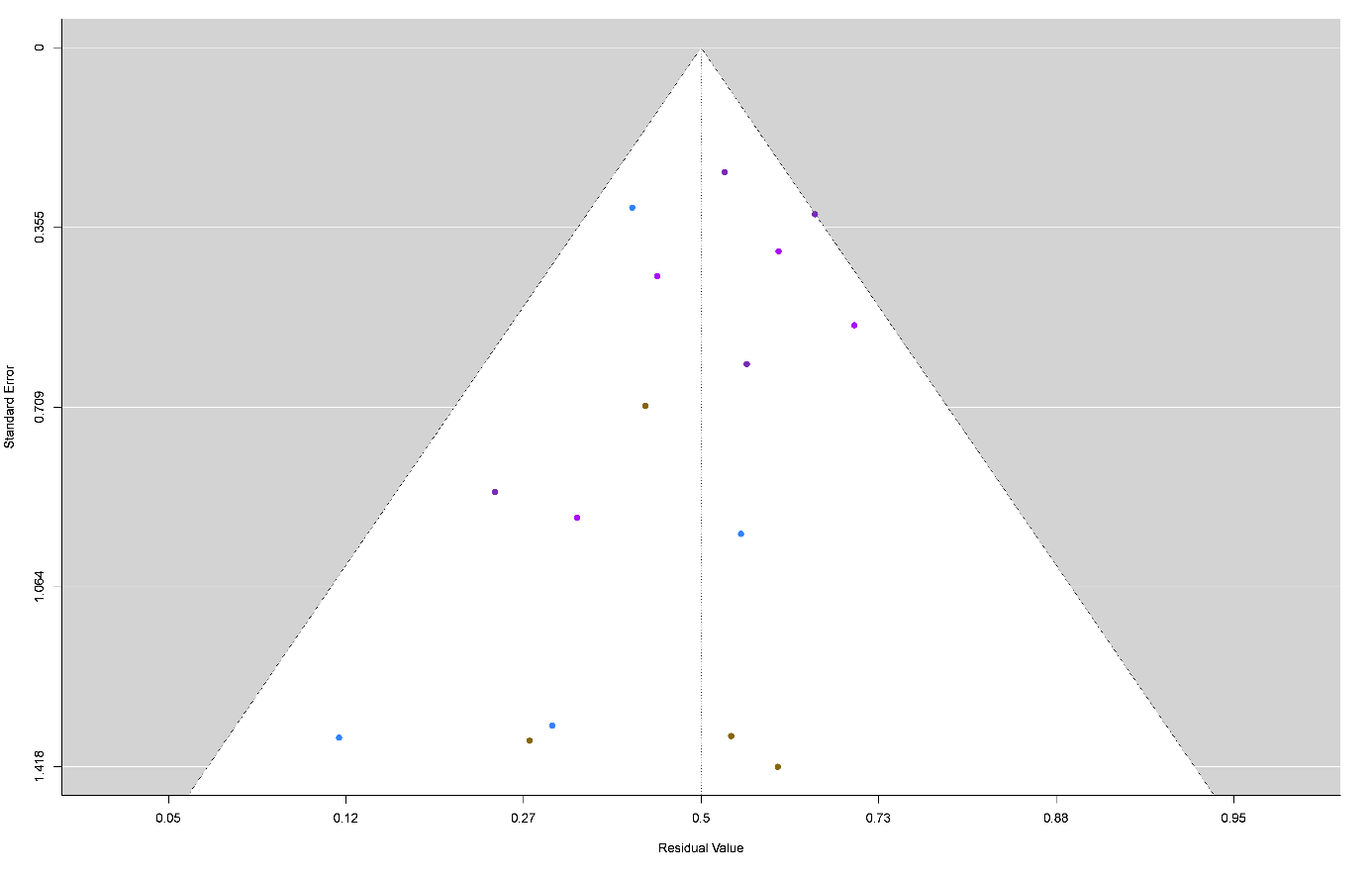


**Supplementary Figure 26.** Funnel plot representing publication bias assessed with Egger's test, in the outcome post-ERCP bleeding in studies using the Haraldsson classification


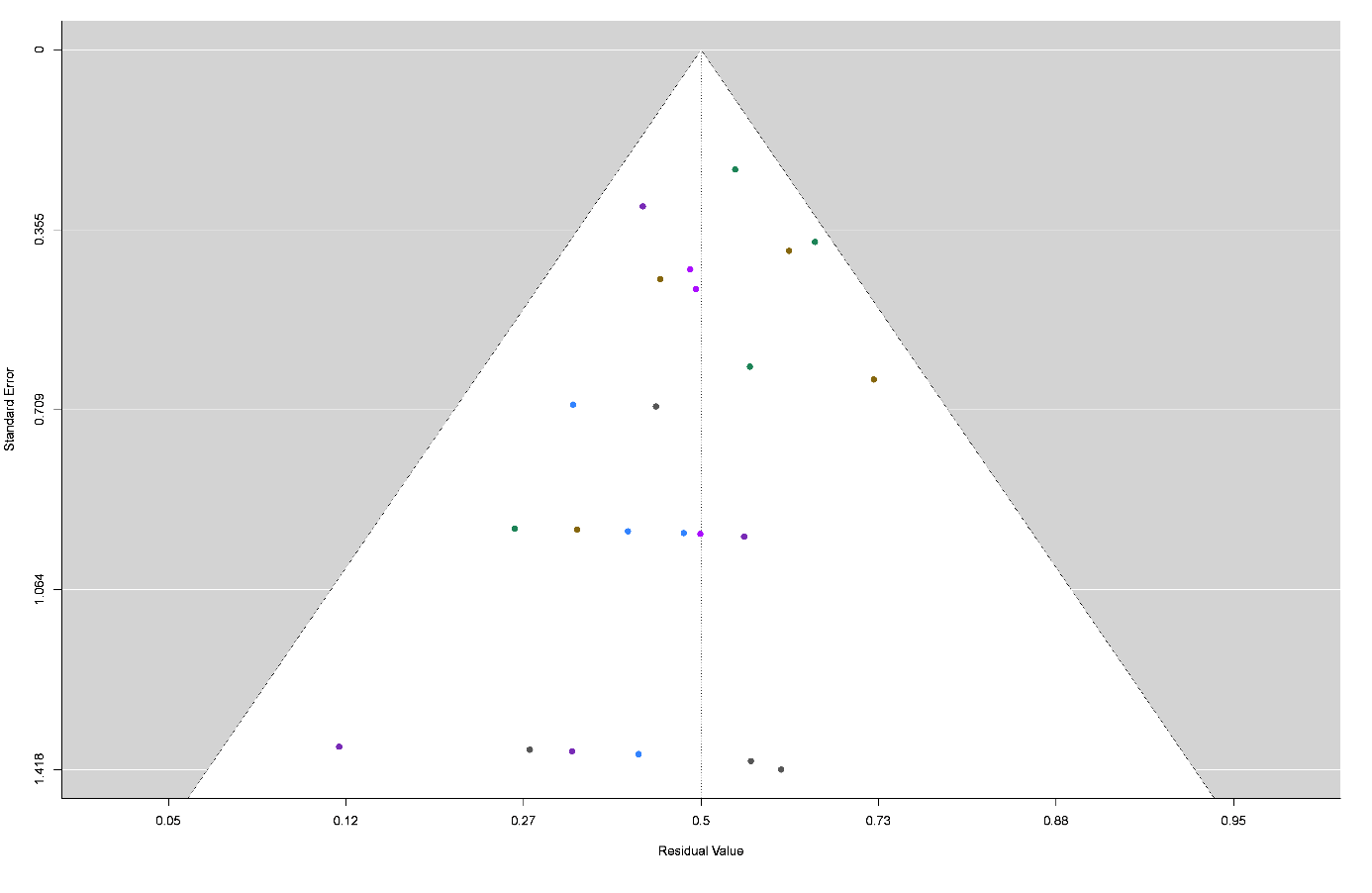


**Supplementary Figure 27.** Funnel plot representing publication bias assessed with Egger's test, in the outcome post-ERCP bleeding in studies using different classification systems

| Embase | ('papilla'/exp OR papilla) AND ('cannulation'/exp OR cannulation OR 'endoscopic retrograde' OR (endoscopic AND retrograde) OR 'ercp'/exp OR ercp) |
| --- | --- |
| Medline | (papilla) AND (cannulation OR endoscopic retrograde OR ERCP) |
| CENTRAL | (papilla) AND (cannulation OR endoscopic retrograde OR ERCP) |

**Supplementary Table 1.** Detailed search strategy

| **Study** | **Classification** | **Number of patients** | **Difficult cannulation (%)** | **Cannulation failure (%)** | **Cannulation time** | **Number of cannulation attempts** |
| --- | --- | --- | --- | --- | --- | --- |
| Balan et al. 2020 | Regular:  Canard type I  Canard type II:  Canard type III:  Canard type IV | 168  36  61  32  25 | 42 (25%)  24 (66.7%)  20 (32.8%)  15 (46.9%)  10 (40%) |  | mean 3.7 min (SD: 2.2)  mean 5.6 min (SD: 2.4)  mean 3.8 min (SD: 2)  mean 3.8 min (SD: 1.7)  mean 4.1 min (SD: 2.1) | median 3.5 (p25-75: 2-4)  median 5.5 (p25-75: 4-7)  median 4 (p25-75: 3-5)  median 4.5 (p25-75: 3-6)  median 4 (p25-75: 3-5) |
| Canena et al. 2021 | Viana type I:  Viana type IIa:  Viana type IIb:  Viana type IIc:  Viana type IIIa:  Viana type IIIb:  Viana type IV: | 47  128  109  35  14  14  14 |  | 3 (6.4%)  4 (3.1%)  9 (8.3%)  0 (0%)  5 (35.7%)  4 (28.6%)  2 (14.3%) | median 14.3 min (p25-75: 7-26)  median 5 min (p25-75: 1-38)  median 8.8 min (p25-75: 7-15)  median 5.5 min (p25-75: 3-12)  median 8 min (p25-75: 7-10)  median 9.5 min (p25-75: 5-14)  median 8.7 min (p25-75: 5-21) |  |
| Chen et al. 2020 | Haraldsson type I:  Haraldsson type II:  Haraldsson type III:  Haraldsson type IV: | 118  25  63  80 |  | 2 (1.7%)  3 (12%)  7 (11.1%)  5 (6.3%) | 0-5 min: 67 p^†^; 5-10 min: 31 p; 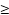10 min: 20 p  0-5 min: 8 p; 5-10 min: 4 p; 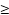10 min: 13 p  0-5 min: 19 p; 5-10 min: 21; 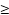10 min: 23 p  0-5 min: 29 p; 5-10 min: 23 p; 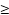10 min: 28 p |  |
| Fernandes et al. 2018 | Leés type I:  Leés type II:  Leés type III:  Leés type IV: | 53  34  13  6 |  |  | median 3.35 min (IQR: 6.84)  median 5.08 min (IQR: 8.53)  median 2.25 min (IQR: 5.66)  median 2.03 min (IQR: 7.51) |  |
| Gutierrez- De Aranguren et al. 2021 | Haraldsson type I:  Haraldsson type II:  Haraldsson type III:  Haraldsson type IV: | 61  46  51  30 | 7 (11.5%)  15 (32.6%)  21 (41.2%)  13 (43.3%) |  |  |  |
| Haraldsson et al. 2019 | Haraldsson type I:  Haraldsson type II:  Haraldsson type III:  Haraldsson type IV: | 771  179  317  110 | 278 (36%)  93 (52%)  152 (48%)  47 (43%) |  | median 139 sec (IQR: 455 sec)  median 269 sec (IQR: 622 sec)  median 245 sec (IQR: 794 sec)  median 210 sec (IQR: 515 sec) |  |
| Liu et al. 2021 | Normal:  Thick and long:  Peridiverticular:  Intradiverticular:  Ectopic:  Edematous:  Ulcerative: | 1198  3025  546  111  1115  196  4899 | 126 (10.5%)  225 (7.4%)  39 (7.1%)  23 (20.7%)  135 (12.1%)  47 (24%)  286 (5.8%) |  |  |  |
| Mohamed et al. 2021 | Haraldsson type I:  Haraldsson type II:  Haraldsson type IIIa:  Haraldsson type IIIb:  Haraldsson type IV:  Haraldsson type D | 392  31  60  58  17  79 |  | 21 (5.4 %)  2 (6.5%)  3 (5%)  7 (12.1%)  2 (11.8%)  6 (7.6%) | mean 5.5 min (SD: 8)  mean 9.7 min (SD: 12.8)  mean 3.3 min (SD: 4.3)  mean 8.6 min (SD: 10.2)  mean 17.8 min (SD: 18.6)  mean 6.1 min (SD: 9.3) | 1-2: 197 p; 3-5: 91 p; 6-10 37 p; >10: 44 p  1-2: 15 p; 3-5: 6 p; 6-10: 4 p; >10: 5 p  1-2: 34 p; 3-5: 20 p; 6-10: 2 p; >10: 3 p  1-2: 24 p; 3-5: 12 p; 6-10: 8 p; >10: 13 p  1-2: 5 p; 3-5: 3 p; 6-10: 2 p; >10: 7 p  1-2: 39 p; 3-5: 10 p; 6-10: 7 p; >10: 13 p |
| Onilla et al. 2021 | Regular protrusion:  Small protrusion:  Large protrusion:  Annular pattern:  Unstructured pattern:  Longitudinal pattern Isolated pattern:  Gyrus pattern: | 196  108  43  250  38  37  3  19 | 70 (35.7%)  50 (46.3%)  17 (39.5%)    88 (35.2%)  21 (55.3%)  16 (43.2%)  2 (66.7%)  10 (52.6%) | 4 (2%)  5 (4.6%)  2 (4.7%)  5 (2%)  3 (7.9%)  2 (5.4%)  0 (0%)  1 (5.3%) |  |  |
| Quiroga-Purizaca et al. 2022 | Haraldsson type I:  Haraldsson type II:  Haraldsson type III:  Haraldsson type IV: | 81  11  40  6 | 19 (23.5%)  4 (36.4%)  17 (42.5%)  3 (50%) |  | average 2.93 min  average 3 min  average 3.67 min  average 6.83 min | average 2.94  average 3.18  average 3.33  average 3.83 |
| Sadeghi et al. 2019 | Small:  Bulging:  Long: | 24  20  28 |  | 6 (25%)  2 (10%)  13 (46.4%) |  |  |
| Saito et al. 2022 | Haraldsson type I:  Haraldsson type II:  Haraldsson type III:  Haraldsson type IV: | 623  618  100  65 | 265 (42.5%)  276 (44.7%)  26 (26%)  33 (50.8%) |  |  |  |
| Thongsuwan et al. 2021 | Haraldsson type I:  Haraldsson type II:  Haraldsson type III:  Haraldsson type IV: | 368  88  69  33 | 60 (16.3%)  25 (28.4%)  16 (23.2%)  9 (27.3%) | 7 (1.9%)  1 (1.2%)  0 (0%)  1 (3%) |  |  |
| Watanabe et al. 2019 | Regular protrusion:  Small protrusion:  Large protrusion:  Annular pattern:  Unstructured pattern:  Longitudinal pattern Isolated pattern:  Gyrus pattern:  Unclassified: | 69  459  61  395  41  44  7  92  10 | 28 (40.6%)  179 (39%)  39 (63.9%)  164 (41.5%)  15 (36.6%)  18 (40.9%)  2 (28.6%)  40 (43.5%)  - | 0 (0%)  11 (2.4%)  3 (4.9%)  8 (2%)  1 (2.4%)  3 (6.8%)  0 (0%)  1 (1.1%)  1 (10%) |  | <5: 41 p; >5: 28 p  <5: 280 p; >5: 179 p  <5: 22 p; >5: 39 p  <5: 231 p; >5: 164 p  <5: 26 p; >5: 15 p  <5: 26 p; >5: 18 p  <5: 5 p; >5: 2 p  <5: 52 p; >5: 40 p  - |
| Zhang et al. 2016 | normal:  small:  bulging:  unusual location: | 18  13  36  15 |  | 0 (0%)  1 (5.6%)  4 (30.8%)  2 (13.3%) | mean 4.1 min (SD: 1.6)  mean 4.7 min (SD: 1.5)  mean 6.6 min (SD: 2.1)  mean 5.1 min (SD: 1.7) |  |

^†^P= number of patients

**Supplementary Table 2.** Systematic review table for outcomes detailing the cannulation process

| **Study** | **Classification** | **Number of patients** | **Post-ERCP**  **pancreatitis (%)** | **Post-ERCP**  **bleeding (%)** | **Post-ERCP**  **perforation (%)** | **Post-ERCP**  **infection (%)** |
| --- | --- | --- | --- | --- | --- | --- |
| Balan et al. 2020 | Regular:  Canard type I  Canard type II:  Canard type III:  Canard type IV: | 168  36  61  32  25 | 18 (10.7%)  5 (13.9%)  2 (2.5%)  1 (3.1%)  7 (28%) | 5 (3%)  0 (0%)  3 (3.7%)  1 (3.1%)  1 (4%) |  | 8 (4.8%)  7 (19.4%)  4 (4.9%)  1 (3.1%)  3 (12%) |
| Canena et al. 2021 | Viana type I:  Viana type IIa:  Viana type IIb:  Viana type IIc:  Viana type IIIa:  Viana type IIIb:  Viana type IV: | 47  128  109  35  14  14  14 | 4 (8.5%)  5 (3.9%)  5 (4.6%)  2 (5.7%)  0 (0%)  2 (14.3%)  0 (0%) | 1 (2.1%)  2 (1.6%)  5 (4.6%)  1 (2.9)  0 (0%)  0 (0%)  0 (0%) | 0 (0%)  0 (0%)  0 (0%)  0 (0%)  0 (0%)  0 (0%)  0 (0%) |  |
| Chen et al. 2020 | Haraldsson type I:  Haraldsson type II:  Haraldsson type III:  Haraldsson type IV: | 118  25  63  80 | 8 (6.8%)  5 (20%)  1 (1.6%)  5 (6.25%) | 6 (5.1%)  2 (8%)  1 (1.6%)  3 (3.8%) | 1 (0.8%)  1 (4%)  0 (0%)  0 (0%) | 3 (2.5%)  0 (0%)  0 (0%)  2 (2.5%) |
| Haraldsson et al. 2019 | Haraldsson type I:  Haraldsson type II:  Haraldsson type III:  Haraldsson type IV: | 771  179  317  110 | 47 (6.1%)  17 (9.4%)  20 (6.4%)  8 (7.5%) |  |  |  |
| Mohamed et al. 2021 | Haraldsson type I:  Haraldsson type II:  Haraldsson type IIIa:  Haraldsson type IIIb:  Haraldsson type IV:  Haraldsson type D: | 392  31  60  58  17  79 | 25 (6.4%)  5 (16.1%)  0 (0%)  7 (12.1%)  2 (11.8%)  4 (5.1%) | 9 (2.3%)  0 (0%)  0 (0%)  0 (0%)  1 (5.9%)  1 (1.3%) |  | 5 (1.3%)  1 (3.2%)  0 (0%)  0 (0%)  0 (0%)  2 (2.5%) |
| Nakeeb et al. 2016 | Normal:  Atrophic:  Pregnant:  Tumour:  Redundant:  Juxtadivertcular:  Small:  Long: | 596  26  70  68  78  84  62  12 | 56 (9.4%)  8 (30.8%)  2 (2.9%)  4 (5.9%)  12 (15.4%)  16 (19%)  2 (3.2%)  2 (16.7%) |  |  |  |
| Quiroga-Purizaca et al. 2022 | Haraldsson type I:  Haraldsson type II:  Haraldsson type III:  Haraldsson type IV: | 81  11  40  6 | 1 (1.2%)  1 (9.1%)  2 (5%)  0 (0%) | 2 (2.5%)  0 (0%)  0 (0%)  0 (0%) | 0 (0%)  1 (9.1%)  0 (0%)  0 (0%) |  |
| Thongsuwan et al. 2021 | Haraldsson type I:  Haraldsson type II:  Haraldsson type III:  Haraldsson type IV: | 368  88  69  33 | 28 (7.7%)  8 (9.2%)  4 (6%)  2 (6.5%) | 14 (3.7%)  1 (1.2%)  4 (6%)  2 (6.5%) |  | 22 (6%)  7 (8.1%)  7 (10.5%)  3 (9.7%) |
| Zheng et al. 2020 | others:  villous:  granular: | 418  1774  193 | 29 (6.9%)  144 (8.1%)  22 (11.4%) |  |  |  |

**Supplementary Table 3.** Systematic review table for outcomes detailing the post-ERCP adverse events

| **N^0^ of studies** | **Certainty assessment** | | | | | | **Effect** | | | **Certainty** | **Importance** |
| --- | --- | --- | --- | --- | --- | --- | --- | --- | --- | --- | --- |
|  | **Study design** | **Risk of bias** | **Inconsistency** | **Indirectness** | **Imprecision** | **Other considerations** | **N^0^ of events** | **N^0^ of individuals** | **Pooled event rate (95% CI)** |  |  |
| type I papilla (assessed with: event rate) | | | | | | | | | | | |
| 5 | observational studies | not serious | very serious^a^ | not serious | serious^b^ | none | - | 1904 | 0.29 (CI. 0.22-0.36) | ⨁◯◯◯ Very low | IMPORTANT |
| type II papilla (assessed with: event rate) | | | | | | | | | | | |
| 5 | observational studies | not serious | very serious^a^ | not serious | serious^b^ | none | - | 942 | 0.41 (CI: 0.32-0.49) | ⨁◯◯◯ Very low | IMPORTANT |
| type III papilla (assessed with: event rate) | | | | | | | | | | | |
| 5 | observational studies | not serious | very serious^a^ | not serious | serious^b^ | none | - | 577 | 0.41 (CI: 0.32-0.50) | ⨁◯◯◯ Very low | IMPORTANT |
| type IV papilla (assessed with: event rate) | | | | | | | | | | | |
| 5 | observational studies | not serious | very serious^a^ | not serious | serious^b^ | none | - | 244 | 0.43 (CI: 0.32-0.55) | ⨁◯◯◯ Very low | IMPORTANT |

**Explanations**

a. wide confidence interval

b. substantial statistical heterogeneity

**Supplementary Table 4.** Summary of findings table for difficult cannulation in studies using the Haraldsson classification

| **N^0^ of studies** | **Certainty assessment** | | | | | | **Effect** | | | **Certainty** | **Importance** |
| --- | --- | --- | --- | --- | --- | --- | --- | --- | --- | --- | --- |
|  | **Study design** | **Risk of bias** | **Inconsistency** | **Indirectness** | **Imprecision** | **Other considerations** | **N^0^ of events** | **N^0^ of individuals** | **Pooled event rate (95% CI)** |  |  |
| type I papilla (assessed with: event rate) | | | | | | | | | | | |
| 8 | observational studies | not serious | very serious^a^ | serious^b^ | serious^c^ | none | - | 2337 | 0.2 (CI. 0.18-0.37) | ⨁◯◯◯ Very low | IMPORTANT |
| type II papilla (assessed with: event rate) | | | | | | | | | | | |
| 8 | observational studies | not serious | very serious^a^ | serious^b^ | serious^c^ | none | - | 1606 | 0.39 (CI: 0.28-0.52) | ⨁◯◯◯ Very low | IMPORTANT |
| type III papilla (assessed with: event rate) | | | | | | | | | | | |
| 8 | observational studies | not serious | very serious^a^ | serious^b^ | serious^c^ | none | - | 713 | 0.35 (CI: 0.25-0.48) | ⨁◯◯◯ Very low | IMPORTANT |
| type IV papilla (assessed with: event rate) | | | | | | | | | | | |
| 5 | observational studies | not serious | very serious^a^ | serious^b^ | serious^c^ | none | - | 244 | 0.41 (CI: 0.28-0.55) | ⨁◯◯◯ Very low | IMPORTANT |

**Explanations**

a. wide confidence interval

b. heterogeneity across the classification systems

c. substantial statistical heterogeneity

**Supplementary Table 5.** Summary of findings table for difficult cannulation in studies using different classifications

| **N^0^ of studies** | **Certainty assessment** | | | | | | **Effect** | | | **Certainty** | **Importance** |
| --- | --- | --- | --- | --- | --- | --- | --- | --- | --- | --- | --- |
|  | **Study design** | **Risk of bias** | **Inconsistency** | **Indirectness** | **Imprecision** | **Other considerations** | **N^0^ of events** | **N^0^ of individuals** | **Pooled event rate (95% CI)** |  |  |
| type I papilla (assessed with: event rate) | | | | | | | | | | | |
| 3 | observational studies | not serious | serious^a^ | not serious | serious^b^ | none | - | 878 | 0.03 (CI. 0.01-0.08) | ⨁⨁◯◯ Low | IMPORTANT |
| type II papilla (assessed with: event rate) | | | | | | | | | | | |
| 3 | observational studies | not serious | serious^a^ | not serious | serious^b^ | none | - | 144 | 0.06 (CI: 0.02-0.18) | ⨁⨁◯◯ Low | IMPORTANT |
| type III papilla (assessed with: event rate) | | | | | | | | | | | |
| 3 | observational studies | not serious | serious^a^ | not serious | serious^b^ | none | - | 250 | 0.07 (CI: 0.03-0.17) | ⨁⨁◯◯ Low | IMPORTANT |
| type IV papilla (assessed with: event rate) | | | | | | | | | | | |
| 3 | observational studies | not serious | serious^a^ | not serious | serious^b^ | none | - | 130 | 0.06 (CI: 0.02-0.17) | ⨁⨁◯◯ Low | IMPORTANT |

**Explanations**

a. wide confidence interval

b. substantial statistical heterogeneity

**Supplementary Table 6.** Summary of findings table for cannulation failure in studies using the Haraldsson classification

| **N^0^ of studies** | **Certainty assessment** | | | | | | **Effect** | | | **Certainty** | **Importance** |
| --- | --- | --- | --- | --- | --- | --- | --- | --- | --- | --- | --- |
|  | **Study design** | **Risk of bias** | **Inconsistency** | **Indirectness** | **Imprecision** | **Other considerations** | **N^0^ of events** | **N^0^ of individuals** | **Pooled event rate (95% CI)** |  |  |
| type I papilla (assessed with: event rate) | | | | | | | | | | | |
| 7 | observational studies | serious | serious^a^ | serious^b^ | not serious | none | - | 1289 | 0.03 (CI. 0.02-0.06) | ⨁◯◯◯ Very low | IMPORTANT |
| type II papilla (assessed with: event rate) | | | | | | | | | | | |
| 7 | observational studies | serious | serious^a^ | serious^b^ | not serious | none | - | 795 | 0.08 (CI: 0.04-0.14) | ⨁◯◯◯ Very low | IMPORTANT |
| type III papilla (assessed with: event rate) | | | | | | | | | | | |
| 7 | observational studies | serious | serious^a^ | serious^b^ | not serious | none | - | 445 | 0.06 (CI: 0.03-0.12) | ⨁◯◯◯ Very low | IMPORTANT |
| type IV papilla (assessed with: event rate) | | | | | | | | | | | |
| 7 | observational studies | serious | serious^a^ | serious^b^ | not serious | none | - | 144 | 0.07 (CI: 0.03-0.17) | ⨁◯◯◯ Very low | IMPORTANT |

**Explanations**

a. wide confidence interval

b. heterogeneity across the classification systems

**Supplementary Table 7.** Summary of findings table for cannulation **failure** in studies using different classifications

| **N^0^ of studies** | **Certainty assessment** | | | | | | **Effect** | | | **Certainty** | **Importance** |
| --- | --- | --- | --- | --- | --- | --- | --- | --- | --- | --- | --- |
|  | **Study design** | **Risk of bias** | **Inconsistency** | **Indirectness** | **Imprecision** | **Other considerations** | **N^0^ of events** | **N^0^ of individuals** | **Pooled event rate (95% CI)** |  |  |
| type I papilla (assessed with: event rate) | | | | | | | | | | | |
| 5 | observational studies | not serious | not serious | not serious | not serious | none | - | 1730 | 0.06 (CI. 0.05-0.08) | ⨁⨁◯◯ Low | IMPORTANT |
| type II papilla (assessed with: event rate) | | | | | | | | | | | |
| 5 | observational studies | not serious | not serious | not serious | not serious | none | - | 334 | 0.11 (CI: 0.08-0.15) | ⨁⨁◯◯ Low | IMPORTANT |
| type III papilla (assessed with: event rate) | | | | | | | | | | | |
| 5 | observational studies | not serious | not serious | not serious | not serious | none | - | 607 | 0.06 (CI: 0.04-0.08) | ⨁⨁◯◯ Low | IMPORTANT |
| type IV papilla (assessed with: event rate) | | | | | | | | | | | |
| 5 | observational studies | not serious | not serious | not serious | not serious | none | - | 246 | 0.07 (CI: 0.04-0.12) | ⨁⨁◯◯ Low | IMPORTANT |

**Supplementary Table 8.** Summary of findings table for post-ERCP pancreatitis in studies using the Haraldsson classification

| **N^0^ of studies** | **Certainty assessment** | | | | | | **Effect** | | | **Certainty** | **Importance** |
| --- | --- | --- | --- | --- | --- | --- | --- | --- | --- | --- | --- |
|  | **Study design** | **Risk of bias** | **Inconsistency** | **Indirectness** | **Imprecision** | **Other considerations** | **N^0^ of events** | **N^0^ of individuals** | **Pooled event rate (95% CI)** |  |  |
| type I papilla (assessed with: event rate) | | | | | | | | | | | |
| 8 | observational studies | serious | not serious | serious^a^ | not serious | none | - | 2622 | 0.07 (CI. 0.06-0.09) | ⨁⨁◯◯ Low | IMPORTANT |
| type II papilla (assessed with: event rate) | | | | | | | | | | | |
| 8 | observational studies | serious | not serious | serious^a^ | not serious | none | - | 540 | 0.10 (CI: 0.07-0.13) | ⨁⨁◯◯ Low | IMPORTANT |
| type III papilla (assessed with: event rate) | | | | | | | | | | | |
| 8 | observational studies | serious | not serious | serious^a^ | not serious | none | - | 744 | 0.05 (CI: 0.04-0.08) | ⨁⨁◯◯ Low | IMPORTANT |
| type IV papilla (assessed with: event rate) | | | | | | | | | | | |
| 6 | observational studies | serious | not serious | serious^a^ | not serious | none | - | 260 | 0.07 (CI: 0.04-0.11) | ⨁⨁◯◯ Low | IMPORTANT |

**Explanations**

a. heterogeneity across the classification systems

**Supplementary Table 9.** Summary of findings table for post-ERCP pancreatitis in studies using different classifications

| **N^0^ of studies** | **Certainty assessment** | | | | | | **Effect** | | | **Certainty** | **Importance** |
| --- | --- | --- | --- | --- | --- | --- | --- | --- | --- | --- | --- |
|  | **Study design** | **Risk of bias** | **Inconsistency** | **Indirectness** | **Imprecision** | **Other considerations** | **N^0^ of events** | **N^0^ of individuals** | **Pooled event rate (95% CI)** |  |  |
| type I papilla (assessed with: event rate) | | | | | | | | | | | |
| 4 | observational studies | not serious | not serious | not serious | not serious | none | - | 1255 | 0.03 (CI. 0.02-0.04) | ⨁⨁◯◯ Low | IMPORTANT |
| type II papilla (assessed with: event rate) | | | | | | | | | | | |
| 4 | observational studies | not serious | not serious | not serious | not serious | none | - | 299 | 0.03 (CI: 0.02-0.07) | ⨁⨁◯◯ Low | IMPORTANT |
| type III papilla (assessed with: event rate) | | | | | | | | | | | |
| 4 | observational studies | not serious | not serious | not serious | not serious | none | - | 357 | 0.03 (CI: 0.01-0.07) | ⨁⨁◯◯ Low | IMPORTANT |
| type IV papilla (assessed with: event rate) | | | | | | | | | | | |
| 4 | observational studies | not serious | not serious | not serious | not serious | none | - | 150 | 0.05 (CI: 0.02-0.10) | ⨁⨁◯◯ Low | IMPORTANT |

**Supplementary Table 10.** Summary of findings table for post-ERCP bleeding in studies using the Haraldsson classification

| **N^0^ of studies** | **Certainty assessment** | | | | | | **Effect** | | | **Certainty** | **Importance** |
| --- | --- | --- | --- | --- | --- | --- | --- | --- | --- | --- | --- |
|  | **Study design** | **Risk of bias** | **Inconsistency** | **Indirectness** | **Imprecision** | **Other considerations** | **N^0^ of events** | **N^0^ of individuals** | **Pooled event rate (95% CI)** |  |  |
| type I papilla (assessed with: event rate) | | | | | | | | | | | |
| 6 | observational studies | serious | not serious | serious^a^ | not serious | none | - | 1255 | 0.07 (CI. 0.06-0.09) | ⨁⨁◯◯ Low | IMPORTANT |
| type II papilla (assessed with: event rate) | | | | | | | | | | | |
| 6 | observational studies | serious | not serious | serious^a^ | not serious | none | - | 299 | 0.10 (CI: 0.07-0.13) | ⨁⨁◯◯ Low | IMPORTANT |
| type III papilla (assessed with: event rate) | | | | | | | | | | | |
| 6 | observational studies | serious | not serious | serious^a^ | not serious | none | - | 357 | 0.05 (CI: 0.04-0.08) | ⨁⨁◯◯ Low | IMPORTANT |
| type IV papilla (assessed with: event rate) | | | | | | | | | | | |
| 5 | observational studies | serious | not serious | serious^a^ | not serious | none | - | 150 | 0.07 (CI: 0.04-0.11) | ⨁⨁◯◯ Low | IMPORTANT |

**Explanations**

a. heterogeneity across the classification systems

**Supplementary Table 11.** Summary of findings table for post-ERCP bleeding in studies using different classifications

| **Section and Topic** | **Item #** | **Checklist item** | **Location where item is reported** |
| --- | --- | --- | --- |
| **TITLE** | | |  |
| Title | 1 | Identify the report as a systematic review. | 1 |
| **ABSTRACT** | | |  |
| Abstract | 2 | See the PRISMA 2020 for Abstracts checklist. | 2 |
| **INTRODUCTION** | | |  |
| Rationale | 3 | Describe the rationale for the review in the context of existing knowledge. | 3 |
| Objectives | 4 | Provide an explicit statement of the objective(s) or question(s) the review addresses. | 3 |
| **METHODS** | | |  |
| Eligibility criteria | 5 | Specify the inclusion and exclusion criteria for the review and how studies were grouped for the syntheses. | 4 |
| Information sources | 6 | Specify all databases, registers, websites, organisations, reference lists and other sources searched or consulted to identify studies. Specify the date when each source was last searched or consulted. | 4 |
| Search strategy | 7 | Present the full search strategies for all databases, registers and websites, including any filters and limits used. | 4, Table S1 |
| Selection process | 8 | Specify the methods used to decide whether a study met the inclusion criteria of the review, including how many reviewers screened each record and each report retrieved, whether they worked independently, and if applicable, details of automation tools used in the process. | 5 |
| Data collection process | 9 | Specify the methods used to collect data from reports, including how many reviewers collected data from each report, whether they worked independently, any processes for obtaining or confirming data from study investigators, and if applicable, details of automation tools used in the process. | 5 |
| Data items | 10a | List and define all outcomes for which data were sought. Specify whether all results that were compatible with each outcome domain in each study were sought (e.g. for all measures, time points, analyses), and if not, the methods used to decide which results to collect. | 5 |
|  | 10b | List and define all other variables for which data were sought (e.g. participant and intervention characteristics, funding sources). Describe any assumptions made about any missing or unclear information. | 5 |
| Study risk of bias assessment | 11 | Specify the methods used to assess risk of bias in the included studies, including details of the tool(s) used, how many reviewers assessed each study and whether they worked independently, and if applicable, details of automation tools used in the process. | 6 |
| Effect measures | 12 | Specify for each outcome the effect measure(s) (e.g. risk ratio, mean difference) used in the synthesis or presentation of results. | 6 |
| Synthesis methods | 13a | Describe the processes used to decide which studies were eligible for each synthesis (e.g. tabulating the study intervention characteristics and comparing against the planned groups for each synthesis (item #5)). | 5-6 |
|  | 13b | Describe any methods required to prepare the data for presentation or synthesis, such as handling of missing summary statistics, or data conversions. | 5-6 |
|  | 13c | Describe any methods used to tabulate or visually display results of individual studies and syntheses. | 5-6 |
|  | 13d | Describe any methods used to synthesize results and provide a rationale for the choice(s). If meta-analysis was performed, describe the model(s), method(s) to identify the presence and extent of statistical heterogeneity, and software package(s) used. | 5-6 |
|  | 13e | Describe any methods used to explore possible causes of heterogeneity among study results (e.g. subgroup analysis, meta-regression). | 6 |
|  | 13f | Describe any sensitivity analyses conducted to assess robustness of the synthesized results. | 6 |
| Reporting bias assessment | 14 | Describe any methods used to assess risk of bias due to missing results in a synthesis (arising from reporting biases). | 6 |
| Certainty assessment | 15 | Describe any methods used to assess certainty (or confidence) in the body of evidence for an outcome. | 6 |
| **RESULTS** | | |  |
| Study selection | 16a | Describe the results of the search and selection process, from the number of records identified in the search to the number of studies included in the review, ideally using a flow diagram. | 6, Figure 1 |
|  | 16b | Cite studies that might appear to meet the inclusion criteria, but which were excluded, and explain why they were excluded. | - |
| Study characteristics | 17 | Cite each included study and present its characteristics. | 6-7, Table 1 |
| Risk of bias in studies | 18 | Present assessments of risk of bias for each included study. | Figures S12-19 |
| Results of individual studies | 19 | For all outcomes, present, for each study: (a) summary statistics for each group (where appropriate) and (b) an effect estimate and its precision (e.g. confidence/credible interval), ideally using structured tables or plots. | 7-9, Figures 2-5, S2, 4, 6, 9 |
| Results of syntheses | 20a | For each synthesis, briefly summarise the characteristics and risk of bias among contributing studies. | 7-9 |
|  | 20b | Present results of all statistical syntheses conducted. If meta-analysis was done, present for each the summary estimate and its precision (e.g. confidence/credible interval) and measures of statistical heterogeneity. If comparing groups, describe the direction of the effect. | 7-9, Figures 2-5, S2, 4, 6, 9 |
|  | 20c | Present results of all investigations of possible causes of heterogeneity among study results. | Figures S1, 3, 5, 7, 8, 10, 11 |
|  | 20d | Present results of all sensitivity analyses conducted to assess the robustness of the synthesized results. | Figures S1, 3, 5, 7, 8, 10, 11 |
| Reporting biases | 21 | Present assessments of risk of bias due to missing results (arising from reporting biases) for each synthesis assessed. | 9-10, Figures S12-19 |
| Certainty of evidence | 22 | Present assessments of certainty (or confidence) in the body of evidence for each outcome assessed. | 10, Tables S4-11 |
| **DISCUSSION** | | |  |
| Discussion | 23a | Provide a general interpretation of the results in the context of other evidence. | 10-12 |
|  | 23b | Discuss any limitations of the evidence included in the review. | 12 |
|  | 23c | Discuss any limitations of the review processes used. | 12 |
|  | 23d | Discuss implications of the results for practice, policy, and future research. | 12-13 |
| **OTHER INFORMATION** | | |  |
| Registration and protocol | 24a | Provide registration information for the review, including register name and registration number, or state that the review was not registered. | 4 |
|  | 24b | Indicate where the review protocol can be accessed, or state that a protocol was not prepared. | 4 |
|  | 24c | Describe and explain any amendments to information provided at registration or in the protocol. | 4 |
| Support | 25 | Describe sources of financial or non-financial support for the review, and the role of the funders or sponsors in the review. | 17 |
| Competing interests | 26 | Declare any competing interests of review authors. | 17 |
| Availability of data, code and other materials | 27 | Report which of the following are publicly available and where they can be found: template data collection forms; data extracted from included studies; data used for all analyses; analytic code; any other materials used in the review. | - |

*From:*  Page MJ, McKenzie JE, Bossuyt PM, Boutron I, Hoffmann TC, Mulrow CD, et al. The PRISMA 2020 statement: an updated guideline for reporting systematic reviews. BMJ 2021;372:n71. doi: 10.1136/bmj.n71

**Supplementary Table 12.** PRISMA 2020 checklist

**Additional details on statistical analyses**

Most articles reported outcome values for all four different papilla morphology (separately for the four types); therefore, we assumed the outcomes were more similar to each other for different samples within the same study than for samples from different studies. Consequently, an additional random effect model was used in our analysis, using a three-level (multi-level) meta-analysis. Inverse variance weighting with a restricted maximum likelihood method was used for pooling the proportions. For confidence interval calculation, a t-distribution method was used. Study heterogeneity was described by the I2 statistics with its 95% CI as given in Cheung et al [1]. We referred to "total I2" for the sum of the two levels as the total heterogeneity over sampling variance. Between and within study heterogeneity, I2 statistics were also given. In the case of 0 cell counts, individual study event rate with 95% CI was calculated by adding 0.5 as continuity correction. For the test of subgroup (papilla type) differences, a "Cochrane Q" test was used [2]. The null hypothesis was rejected at a 5% significance level.

As sensitivity analyses on the multi-level results, we calculated the effect size estimates with 95% CI, dfbetas, Cook’s distances and hat values with two ways: leaving out one record (it means that if a study reports result for more subgroup (papilla types), only the values from one subgroup left out) or leaving out one study.

We provided here only the forest plot where the estimates with confidence interval is given if leaving-out a given study as we think this is the most informative and we found no relevant outlier regarding to the other parameters.

Moreover, we compared the multi-level model AIC and BIC values with two level model with the same calculation method. Furthermore, as the studies have relatively different sample size and in a few cases they report 0 events, we used a random intercept logistic regression model (aka. glmm, with the logit transformation of the observed proportions) as a sensitivity analysis, but here we assumed no “more similarity in the results of the same study”. To estimate the heterogeneity variance measure, a maximum likelihood method was used. We found no relevant difference in information criteria values nor with the comparison with the glmm model. (We did not included these results.)

1 Cheung, M. W. Modeling dependent effect sizes with three-level meta-analyses: a structural equation modeling approach. *Psychol Methods* **19**, 211-229, doi:10.1037/a0032968 (2014).

2 Harrer, M., Pim Cuijpers, Furukawa Toshi A, and David D Ebert. 2021. Doing Meta-Analysis With R: A Hands-On Guide. 1st ed. Boca Raton, FL; London: Chapman & Hall/CRC Press.

.
